# A digital shadow of CAR T cell expansion in a perfusion bioreactor: Informing optimal harvest times for autologous cell therapy

**DOI:** 10.1002/btpr.70045

**Published:** 2025-06-23

**Authors:** Joseph R. Egan, Núria Marí‐Buyé, Elia Vallejo Benítez‐Cano, Miquel Costa, Linda Wanika, Michael J. Chappell, Ursula Schultz, Jelena Ochs, Manuel Effenberger, David Horna, Qasim Rafiq, Stephen Goldrick

**Affiliations:** ^1^ Department of Biochemical Engineering University College London London UK; ^2^ School of Health and Life Sciences, National Horizons Centre Teesside University Darlington UK; ^3^ Aglaris Cell Tres Cantos Spain; ^4^ Aglaris Ltd. Stevenage UK; ^5^ School of Engineering University of Warwick Coventry UK; ^6^ Sartorius CellGenix GmbH Freiburg Germany

**Keywords:** cell growth, mathematical model, PID control, predictive analytics, predictive model, process analytical technology, process model, soft sensor

## Abstract

Chimeric antigen receptor (CAR) T cell therapy has tremendous potential for the treatment of cancer and other diseases. To manufacture cells of the desired quantity and quality, it is important to expand the CAR T cells ex vivo for an optimal duration. However, identifying the optimal harvest time requires knowledge of the cell concentration during the expansion period. To address this challenge, we have developed a digital shadow of CAR T cell expansion that provides a soft sensor of cell concentration in real‐time. Specifically, a novel mechanistic mathematical model of cell growth within a proportional‐integral‐derivative (PID) controlled perfusion bioreactor has been developed using nonlinear ordinary differential equations. The model is fitted to data generated via bioreactor runs of the Aglaris FACER, in which both donor and patient cells have been expanded in two different media. Off‐line data includes the initial and final cell concentrations, and online data includes the glucose and lactate concentrations as well as the perfusion rate. Training the digital shadow utilizes all the off‐line and online data for each run. In contrast, real‐time testing utilizes only the initial cell concentration and the available online data at the time of model fitting. Real‐time testing shows that with at least 2.5 days of online data, the final cell concentration up to 2.5 days later is predicted with a mean relative error of 13% (standard deviation ≈ 6%). Informative real‐time predictions of cell concentration via the digital shadow can guide decisions regarding the optimal harvest time of CAR T cells.

## Graphical abstract

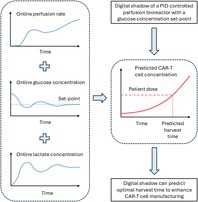

Using online metabolite data from the first few days of a bioreactor run, the digital shadow can forecast the cell concentration in real‐time with an accuracy of 13% (standard deviation ≈ 6%). These predictions can guide the optimal harvest time of CAR T cells for subsequent re‐infusion into the patient.

## 1 INTRODUCTION

In the past decade, chimeric antigen receptor (CAR) T cell therapy has had considerable success in the treatment of relapsed/refractory hematological malignancies, including large B cell lymphoma and multiple myeloma.^1^, ^2^, ^3^ As of November 2024, there have been seven autologous CAR T cell therapies approved by the US Food and Drug Administration (FDA)^3^, ^4^ and there are hundreds of ongoing clinical trials not just against a variety of cancers but also increasingly aimed at autoimmune, infectious, and other diseases (see clinicaltrials.gov). However, treatment costs are currently of the order of hundreds of thousands of US dollars per patient, a significant proportion of which is associated with the multi‐stage manufacturing process.^4^, ^5^ A critical period in the manufacture of CAR T cells is the expansion (or growth/proliferation) of the patient's gene‐modified cell population.^6^ Conventional expansion periods can range from 1 to 2 weeks^4^, ^7^, ^8^ and so the possibility of shortening this stage potentially offers significantly reduced manufacturing costs. In addition, reduced expansion times have the advantage of allowing shorter waiting periods for patients who could be experiencing rapidly advancing disease.^5^, ^9^ Furthermore, prolonged expansion has been associated with progressive differentiation of CAR T cells that can result in less effective treatment.^8^, ^9^, ^10^, ^11^, ^12^, ^13^, ^14^, ^15^ Indeed, one study found that reducing a standard 9‐day expansion to 3 or 5 days could improve CAR T cell functionality.^16^ On the other hand, for activated CAR T cells to be an effective therapeutic against cancer, they must be administered to a patient at a sufficiently high dose of cells.^6^, ^14^, ^15^, ^17^, ^18^, ^19^ Reaching such dose thresholds with shortened expansion times might not be possible for patients who have gone through prior lymphodepleting cancer treatment.^5^, ^7^ Taken together, this suggests that both the quality as well as the quantity of CAR T cells are critical quality attributes (CQAs) of the manufacturing process^10^, ^19^ and that maximizing any quality vs. quantity trade‐off might be achieved via an optimal period of CAR T cell expansion.

The personalized nature of autologous CAR T cell therapy means that an optimal expansion period is likely to be both patient and product specific and, therefore, the expansion period should ideally not be set at the same value for every manufactured batch. Instead, cell growth related information obtained in real‐time could help to inform the human operator regarding the optimal time at which to terminate expansion for subsequent cryopreservation or reinfusion into the patient (i.e., the optimal harvest time^10^, ^18^, ^20^). To gain such situational awareness, a variety of process analytical technologies (PATs) could be used to measure cell concentration during expansion in a bioreactor.^20^, ^21^ For example, spectroscopic techniques including dielectric spectroscopy (bio‐capacitance) can, when calibrated, give accurate readings of cell concentration.^22^, ^23^ These so‐called ‘hard sensors’ are particularly suited to cells in suspension, such as when cells are grown in stirred tank bioreactors^18^, ^24^, ^25^, ^26^, ^27^ where space is available to insert additional probes. However, they are less suited to bioreactors where the cells grow whilst sedimented on a membrane, especially in small‐scale bioreactors designed for autologous cell therapy.^28^ In such cases, alternative methods can be used to gain insight into the expanding cell population. One approach is to resuspend the cells and take a sample which can then be assessed off‐line for cell quantity and quality via a cell counter and flow cytometry, respectively. However, this manual approach risks breaching the sterility barrier,^18^, ^20^, ^26^, ^29^ is time‐consuming, and can be potentially deleterious to continued cell growth. As such, taking samples in this manner would ideally be limited to the end of the expansion period for quality assurance purposes only, rather than continually repeated throughout the bioreactor run. Although the above‐mentioned hard sensors may not be appropriate to provide a direct measure of cell concentration, it may instead be possible to utilize so‐called ‘soft sensors’^22^, ^30^, ^31^, ^32^ to provide an indirect prediction of cell growth. Such soft sensors could combine other hard‐sensor measurements of a variety of critical process parameters (CPPs, such as pH, dissolved oxygen, nutrient/metabolite concentrations^15^) with mechanistic and/or data‐driven models to provide predictions of cell growth in real‐time.

Whether cells are grown in suspension or sedimentation, the process of perfusion (whereby ‘fresh’ media is continually fed into the bioreactor and “spent media” is continually removed) provides conditions that allow for improved cell quantity and quality when compared to alternative processes such as fed‐batch or ‘perfusion‐mimic’ (i.e. media exchange at fixed time‐points^24^, ^25^, ^27^, ^33^).^10^, ^22^, ^34^ Bioreactors on the CAR T cell market that operate via perfusion^5^, ^10^, ^21^, ^35^ generally utilize a fixed perfusion rate for the duration of the expansion or utilize perfusion rates that are adjusted either manually or based on a pre‐defined protocol.^26^, ^36^, ^37^ Such feeding regimes are often based on either prior knowledge of the process or daily samples of nutrients and metabolites. However, these strategies generally do not consider the variable batch‐to‐batch needs resulting from patient‐to‐patient variability, or at least their feeding protocols are not optimized to respond to such variability.^26^, ^29^, ^36^ Thus, for autologous CAR T cell therapy, an optimal perfusion rate is one that varies with time depending on the requirements of the cells.^10^, ^26^ This means that both the optimal time‐varying perfusion rate and the optimal duration of expansion are patient specific; indeed, to truly achieve the latter, the former must first be met.

In broad terms, a digital twin can be considered a virtual representation of a physical system in which there is a two‐way transfer of data between the physical and the virtual components.^22^, ^28^, ^38^, ^39^, ^40^, ^41^ Such two‐way data transfer means that a digital twin can adaptively control the physical system based on continuously updated calculations of the virtual component. In contrast, a digital shadow has a one‐way transfer of data from the physical to the virtual component and, therefore, adaptive control is not possible.^22^, ^38^, ^39^, ^40^, ^41^ However, by receiving data in real‐time a digital shadow can still provide quantitative insight into the system's processes. In this paper, we develop a digital shadow of cell growth in a perfusion bioreactor designed for autologous cell therapy. Specifically, the digital shadow is applicable to bioreactors in which a proportional‐integral‐derivative (PID) controller is used to maintain the glucose concentration at a set point by frequently adjusting the perfusion rate. By utilizing high‐frequency online data of the perfusion rate, the glucose concentration and the lactate concentration, the digital shadow can provide ‘nowcasts’ of the cell concentration (i.e. frequently updated predictions of the cell concentration during the bioreactor run) as well as a short‐term forecast a few days into the future. Thus, our digital shadow provides a novel soft sensor of cell concentration that can be used to guide the patient‐specific period of CAR T cell expansion. This soft sensor is particularly useful for perfusion bioreactors in which cell retention is achieved via sedimentation because of the challenge of measuring cell concentration via hard sensors and the disturbance to cell growth that occurs when resuspending the cells to take samples for offline analysis.

To illustrate its application, the digital shadow is initially fitted to five runs of the FACER—a bioreactor developed by Aglaris Ltd. over the past decade. After being trained on these data, the digital shadow is then tested against the same datasets in a pseudo‐real‐time environment (i.e. as the data would have been received in real‐time). In addition, the digital shadow is also tested against an independent FACER training dataset not used in the original training. We show that with knowledge of the initial cell concentration and at least 2.5 days of online data, the digital shadow can provide predictions of the cell concentration up to 2.5 days into the future with a mean relative error of 13%. Such real‐time predictions can be used to inform the optimal harvest time of CAR T cells for subsequent patient therapy.

## 2 MATERIALS AND METHODS

### 2.1 Mathematical

**FIGURE 1:**
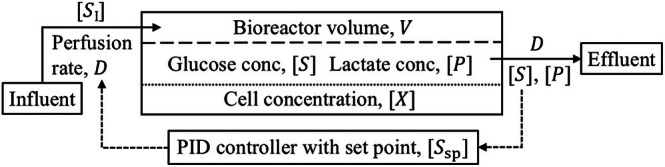
Schematic of a proportional‐integral‐derivative (PID) controlled perfusion bioreactor where cell retention is via sedimentation. Solid arrows represent media flow and dashed arrows represent PID feedback where glucose concentration, $\left[S\right]$ is the process variable and perfusion rate, D is the control variable.

Figure 1 shows a schematic of the modeled cell expansion process whereby the cells, with concentration denoted by $\left[X\right]$, are retained in the bioreactor via sedimentation. Once a very high proportion of the cells have sedimented, ‘fresh media', with a glucose concentration denoted by $\left[S_{I}\right]$, is perfused into the bioreactor at a rate D. At the same time “spent media,” with glucose and lactate concentrations denoted by $\left[S\right]$ and $\left[P\right]$, respectively, is perfused out of the bioreactor at the same rate D, thus maintaining a constant volume, denoted by V. The perfusion rate is continually adjusted by a proportional‐integral‐derivative (PID) controller that acts to maintain the glucose concentration at a set‐point, $\left[S_{\mathrm{sp}}\right]$. Therefore, the PID controller requires online data of the perfusion rate D and glucose concentration $\left[S\right]$.

The key equations of the digital shadow and the fitting process are outlined below. Further details can be found in the Supporting Information. To be clear, the perfusion rate, $D\left(t\right)$ is equivalent to:

(1) $$D\left(t\right)=Q\left(t\right)/V$$

where $Q\left(t\right)$ denotes the volumetric flow rate of the bioreactor.^34^ It is also worth clarifying that the perfusion rate is often given in terms of vessel volume per day (VVD).^33^ For example, $D\left(t\right)=1$/day can be interpreted as a volume of media equivalent to the volume of 1 bioreactor vessel passing through the bioreactor per day.

As cell retention is via sedimentation, in the physical bioreactor cells are not homogeneously distributed in the media. However, for simplicity, the contents of the bioreactor are modeled as homogeneous (i.e., the fresh/spent media and cells are modeled as well mixed). As such, the model is of the unstructured and unsegregated type^41^ and is formulated via nonlinear ordinary differential equations (ODEs). Also for simplicity, we model the perfusion rate as a proportional‐integral (PI) controller with parameters $K_{p}$ and $K_{i}$. This is a good approximation to a proportional‐integral‐derivative (PID) controller if the derivative term is having a negligible impact. As such, the ODE for the perfusion rate is given by:

(2) $$\frac{\mathrm{dD}}{\mathrm{dt}}=H\left(t-t_{\mathrm{sed}}\right)\left(-K_{p}\frac{d\left[S\right]}{\mathrm{dt}}+K_{i}\left(\left[S_{\mathrm{sp}}\right]-\left[S\left(t\right)\right]\right)\right)\;\text{for}\;\;t\geq t_{I}$$

where t denotes time, $t_{I}$ denotes the initial time when the cells are seeded in the bioreactor and $t_{\mathrm{sed}}$ denotes the time at which the sedimentation period ends (and when perfusion starts). H denotes the Heaviside step function which, here, is equal to 0 during the sedimentation period (denoted by $t_{\mathrm{sed}}-t_{I}$) and equal to 1 during the perfusion period (denoted by $t_{F}-t_{I}$). Note that an equivalent equation for the perfusion rate based on a PID controller is provided in section 2 of the Supporting Information.

ODEs for the glucose concentration and lactate concentration are modeled as functions of the influent and effluent of the perfusion, as well as the cells consumption and production, respectively, given by:

(3) $$\frac{d\left[S\right]}{\mathrm{dt}}=H\left(t-t_{\mathrm{sed}}\right)D\left(t\right)\left(\left[S_{I}\right]-\left[S\left(t\right)\right]\right)-\frac{\mu \left(t\right)\left[X\left(t\right)\right]}{Y_{\mathrm{xs}}}\;\text{for}\;t\geq t_{I}$$

(4) $$\frac{d\left[P\right]}{\mathrm{dt}}=Y_{\mathrm{px}}\mu \left(t\right)\left[X\left(t\right)\right]-H\left(t-t_{\mathrm{sed}}\right)D\left(t\right)\left(\left[P\left(t\right)\right]\right)\;\text{for}\;t\geq t_{I}$$

where $Y_{\mathrm{xs}}$ and $Y_{\mathrm{px}}$ denote the yield of cells from glucose, and the yield of lactate from cells, respectively. Note that the yield of lactate from glucose, $Y_{\mathrm{ps}}$ is given by the product of the constituent yields as follows:

(5) $$Y_{\mathrm{ps}}=Y_{\mathrm{px}}Y_{\mathrm{xs}}$$

Let $\left[C\right]$ denote the dissolved oxygen (DO) concentration in the bioreactor. A typical ODE to model the DO concentration is given by:

(6) $$\frac{d\left[C\right]}{\mathrm{dt}}=\left(H\left(t-t_{\mathrm{sed}}\right)D\left(t\right)+k_{L}a\right)\left(\left[C^{*}\right]-\left[C\left(t\right)\right]\right)-\frac{\mu \left(t\right)\left[X\left(t\right)\right]}{Y_{\mathrm{xc}}}\;\text{for}\;t\geq t_{I}$$

where $\left[C^{*}\right]$ is the saturated DO percentage, $k_{L}a$ is the volumetric mass transfer coefficient, and $Y_{\mathrm{xc}}$ denotes the yield of cells from DO.^34^ On the right‐hand‐side of Equation (6) the first term is often referred to as the oxygen transfer rate (OTR) and the second term as the oxygen uptake rate (OUR).^42^ Note that Equation (3) models fresh media being provided to the cells during the perfusion period alone whereas Equation (6) models fresh gas being supplied to the cells throughout the entire process. DO data are often reported as a percentage or a proportion of its saturated value.^34^ In such circumstances Equation (6) can be adapted for model fitting purposes by first letting $C^{\prime }\left(t\right)$ denote the normalized DO (i.e., the DO concentration scaled by its saturated value) given by:

(7) $$C^{\prime }\left(t\right)=\left[C\left(t\right)\right]/\left[C^{*}\right]$$

Substituting Equation (7) into Equation (6) gives:

(8) $$\frac{{\mathrm{dC}}^{\prime }}{\mathrm{dt}}=\left(H\left(t-t_{\mathrm{sed}}\right)D\left(t\right)+k_{L}a\right)\left(1-C^{\prime }\left(t\right)\right)-\frac{\mu \left(t\right)\left[X\left(t\right)\right]}{Y\prime_{\mathrm{xc}}}\;\text{for}\;t\geq t_{I}$$

where:

(9) $$Y\prime_{\mathrm{xc}}=Y_{\mathrm{xc}}\left[C^{*}\right]$$

$\left[X\left(t\right)\right]$ and $\mu \left(t\right)$ are the cell concentration and specific growth rate, respectively, which are modeled by a lag period, denoted by $t_{\mathrm{lag}}-t_{I}$, followed by a special case of the Richards growth model^43^ in the early stages of cell growth, given by:

(10) Xt=XI1−Ht−tlagμmaxνt−tlag1νfort≥tIandν<0

(11) $$\mu \left(t\right)=\frac{H\left(t-t_{\mathrm{lag}}\right)\mu_{\max}}{1-H\left(t-t_{\mathrm{lag}}\right)\mu_{\max}\nu \left(t-t_{\mathrm{lag}}\right)}\;\text{for}\;t\geq t_{I}\;\text{and}\;\nu <0$$

where $\left[X_{I}\right]$ is the initial cell concentration at time $t_{I}$, $\mu_{\max}$ is the maximum specific growth rate at time $t_{\mathrm{lag}}$ and ν is a parameter that determines the decrease of the specific growth rate over time. Again, H denotes the Heaviside step function which, here, is equal to 0 during the lag period and equal to 1 when the cells start growing (i.e., for $t\geq t_{\mathrm{lag}}$). Note that in Equations (10) and (11), H is a function of time whereas ν is not (i.e., $\nu \left(t-t_{\mathrm{lag}}\right)$ denotes that ν is multiplied by $\left(t-t_{\mathrm{lag}}\right)$). To the best of our knowledge, Equations (10) and (11) are novel derivations.

If the final cell concentration, $\left[X_{F}\right]$ at time $t_{F}$ is known then $\mu_{\max}$ can be given as a combination of the other parameters via Equation (10):

(12) $$\mu_{\max}=\frac{1-{\left(\left[X_{I}\right]/\left[X_{F}\right]\right)}^{\nu }}{\nu \left(t_{F}-t_{\mathrm{lag}}\right)}\;\text{for}\;t_{F}>t_{\mathrm{lag}}\;\text{and}\;\nu <0$$

With an analysis of a complete bioreactor dataset, Equation (12) allows one less parameter to be fitted compared with a real‐time analysis. Equation (10) can also be rearranged to give the time, $t_{T}$ at which the cell concentration reaches a threshold concentration, $\left[X_{T}\right]$:

(13) $$t_{T}=t_{\mathrm{lag}}+\frac{1-{\left(\left[X_{I}\right]/\left[X_{T}\right]\right)}^{\nu }}{\mu_{\max}\nu }\;\text{for}\;t_{T}>t_{\mathrm{lag}}\;\text{and}\;\nu <0$$

Depending on the threshold cell concentration required for clinical use (i.e., the required patient dose), Equation (13) can be used to predict the harvest time of the cells (or the time at which to take a confirmatory suspension sample to perform an off‐line measurement of the cell concentration) once the parameters $t_{\mathrm{lag}}$, $\mu_{\max}$, and ν have been fitted to the available data.

Let it be assumed that at time $t_{\mathrm{sp}}>\left\{t_{\mathrm{sed}},t_{\mathrm{lag}}\right\}$ the glucose concentration remains approximately at its set‐point via the PI(D) controller adjusting the perfusion rate appropriately. In this scenario:

(14) $$\frac{d\left[S\right]}{\mathrm{dt}}\approx 0\;\text{for}\;t\geq t_{\mathrm{sp}}$$

Substituting Equations (10), (11), and (14) into Equation (3) and rearranging gives:

(15) $$D_{\mathrm{sp}}\left(t\right)=\frac{\mu_{\max}\left[X_{I}\right]}{Y_{\mathrm{xs}}\left(\left[S_{I}\right]-\left[S_{\mathrm{sp}}\right]\right){\left(1-\mu_{\max}\nu \left(t-t_{\mathrm{lag}}\right)\right)}^{\frac{1+\nu }{\nu }}}\;\text{for}\;t\geq t_{\mathrm{sp}}\;\text{and}\;\nu <0$$

where $D_{\mathrm{sp}}\left(t\right)$ is the approximate perfusion rate once the glucose concentration has stabilized at its set‐point. Combining Equations ((3), (4), (5))–((3), (4), (5)) and (14) gives:

(16) $$\frac{d\left[P\right]}{\mathrm{dt}}=D_{\mathrm{sp}}\left(t\right)\left(Y_{\mathrm{ps}}\left(\left[S_{I}\right]-\left[S_{\mathrm{sp}}\right]\right)-\left[P\left(t\right)\right]\right)\;\text{for}\;t\geq t_{\mathrm{sp}}$$

Equation (16) is variables‐separable and can be integrated with respect to time to give:

(17) $$\left[P\left(t\right)\right]=Y_{\mathrm{ps}}\left(\left[S_{I}\right]-\left[S_{\mathrm{sp}}\right]\right)-\left(Y_{\mathrm{ps}}\left(\left[S_{I}\right]-\left[S_{\mathrm{sp}}\right]\right)-\left[P\left(t_{\mathrm{sp}}\right)\right]\right)\exp\left(-\int_{t_{\mathrm{sp}}}^{t}D_{\mathrm{sp}}\mathrm{dt}\right)\text{for}\;t\geq t_{\mathrm{sp}}$$

It can be seen from Equation (17) that $\left[P\left(t\right)\right]\to \left[P_{\mathrm{ss}}\right]$ as t→∞ where:

(18) $$\left[P_{\mathrm{ss}}\right]=Y_{\mathrm{ps}}\left(\left[S_{I}\right]-\left[S_{\mathrm{sp}}\right]\right)$$

Equation (17) shows that if the glucose concentration remains at its set‐point via control of the perfusion rate, then the lactate concentration will tend to a steady state given by Equation (18). The simplicity of Equation (18) is noteworthy; it shows that the lactate concentration steady state is simply the yield of lactate from glucose multiplied by the difference between the glucose concentration in the fresh media and the glucose concentration set‐point.

### 2.2 Experimental

**FIGURE 2:**
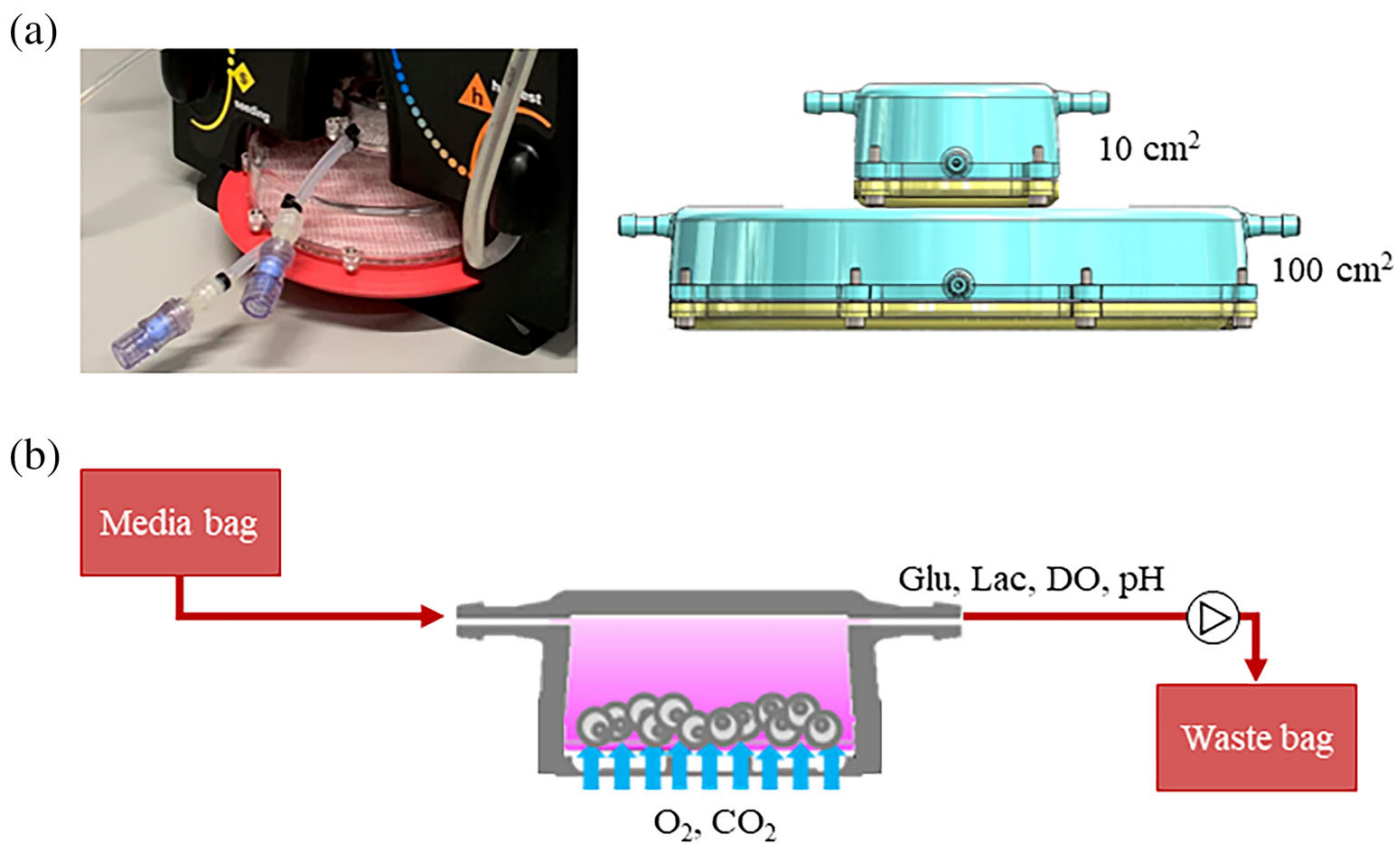
The FACER culture system. (a) Photograph and illustration of the two culture chambers within the FACER single‐use cartridge where numbers refer to the surface area available to the cells in each chamber. (b) Schematic of the cell culture within the chambers depicting the flow of medium inlet and outlet, gas feeding through the membrane, and online measurements of glucose and lactate concentrations, dissolved oxygen, and pH.

The cell expansion process shown in Figure 1 is applicable to the Aglaris FACER, a completely closed and automated cell production system designed to work in a GMP environment to produce advanced therapies. Suspension cells, such as T cells, can be activated, transduced, expanded, washed, and formulated within the platform. The single‐use cartridge consists of two culture chambers where the cells grow, alongside all the tubing, connectors, valves, and single‐use sensors necessary for automated processing and cell monitoring (see Figure 2). Within the culture chambers, cells are cultivated under static conditions, promoting cell–cell contact and minimizing shear stress. The chambers provide two levels of surface (with volumes of 13.4 and 134 mL and surface areas of 10 and 100 cm^2^, respectively) to accommodate the cells in their optimal concentration range during the whole process. This flexibility enables the production of therapeutic doses from varying starting cell numbers. Thus, when the starting cell number ranges from ~5 to 20 million cells, the cells are inoculated in the small chamber and later passaged to the large chamber. If the starting cell number ranges from ~50 to 200 million cells, the cells are directly seeded in the large chamber. In both chambers, the controlled media zone is separated from the controlled gas zone with a gas‐permeable membrane, on which the cells are cultured.

The single‐use cartridge also includes circuitry and sensors that enable process automation. The cell expansion process is closely monitored by online single‐use sensors that provide high‐frequency readings of CPPs such as pH, DO, and glucose and lactate concentrations. Specifically, glucose and lactate concentrations are measured every 10 s via enzymatic sensors. In addition to the online measurements, accurately measuring the cell concentration requires the cells to be in suspension. However, resuspending the cells after they have sedimented can disturb their growth and, therefore, suspension samples are only taken prior to sedimentation and when perfusion has ended. Sampling for off‐line analysis can be conducted in two ways: from suspended cells for both cell count and phenotype characterization or from sedimented cells, which does not allow for cell count but provides a relevant sample for phenotype characterization.

**FIGURE 3:**
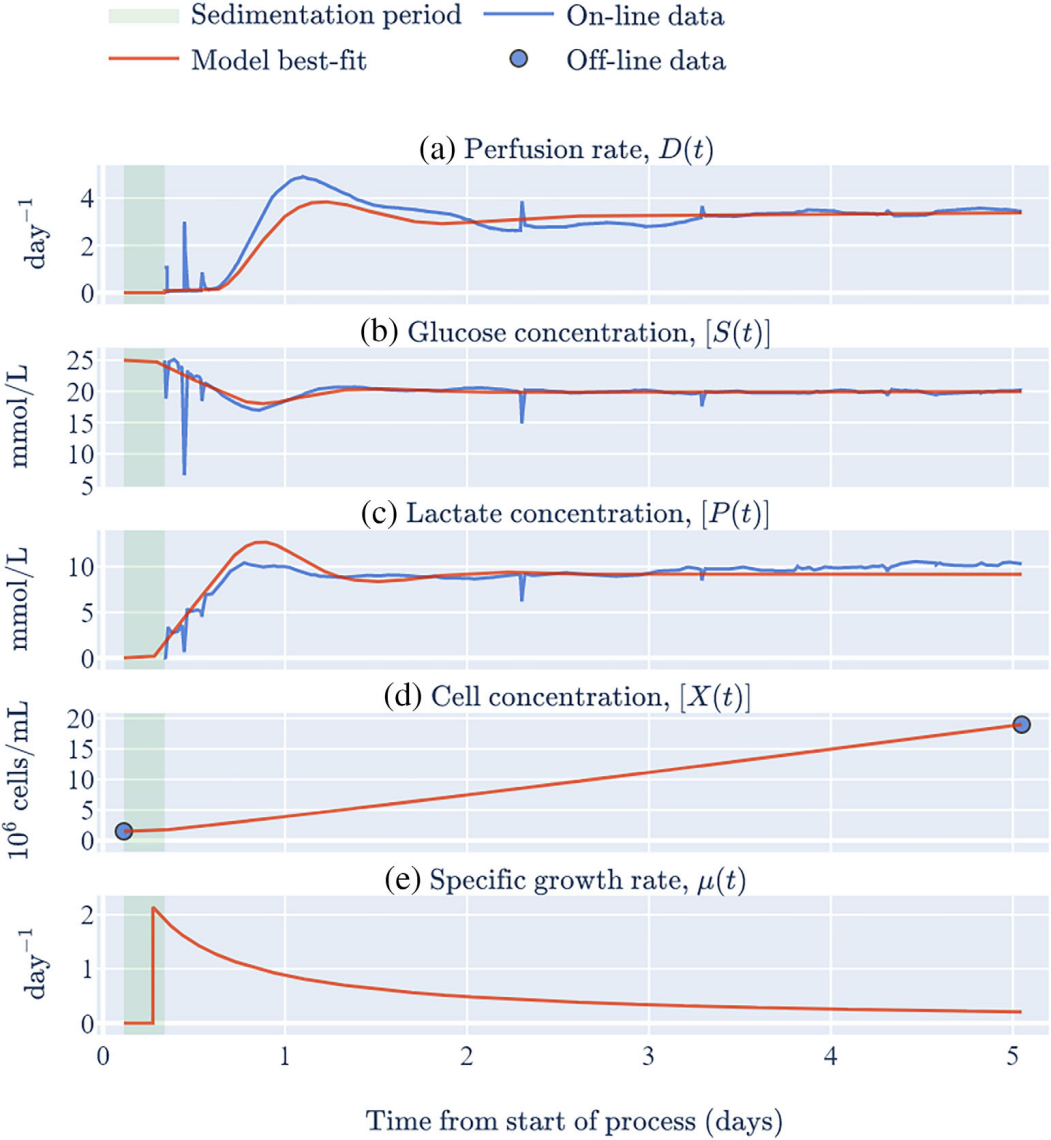
Key data from run 1 in the large chamber of the Aglaris FACER and model best‐fits.

**FIGURE 4:**
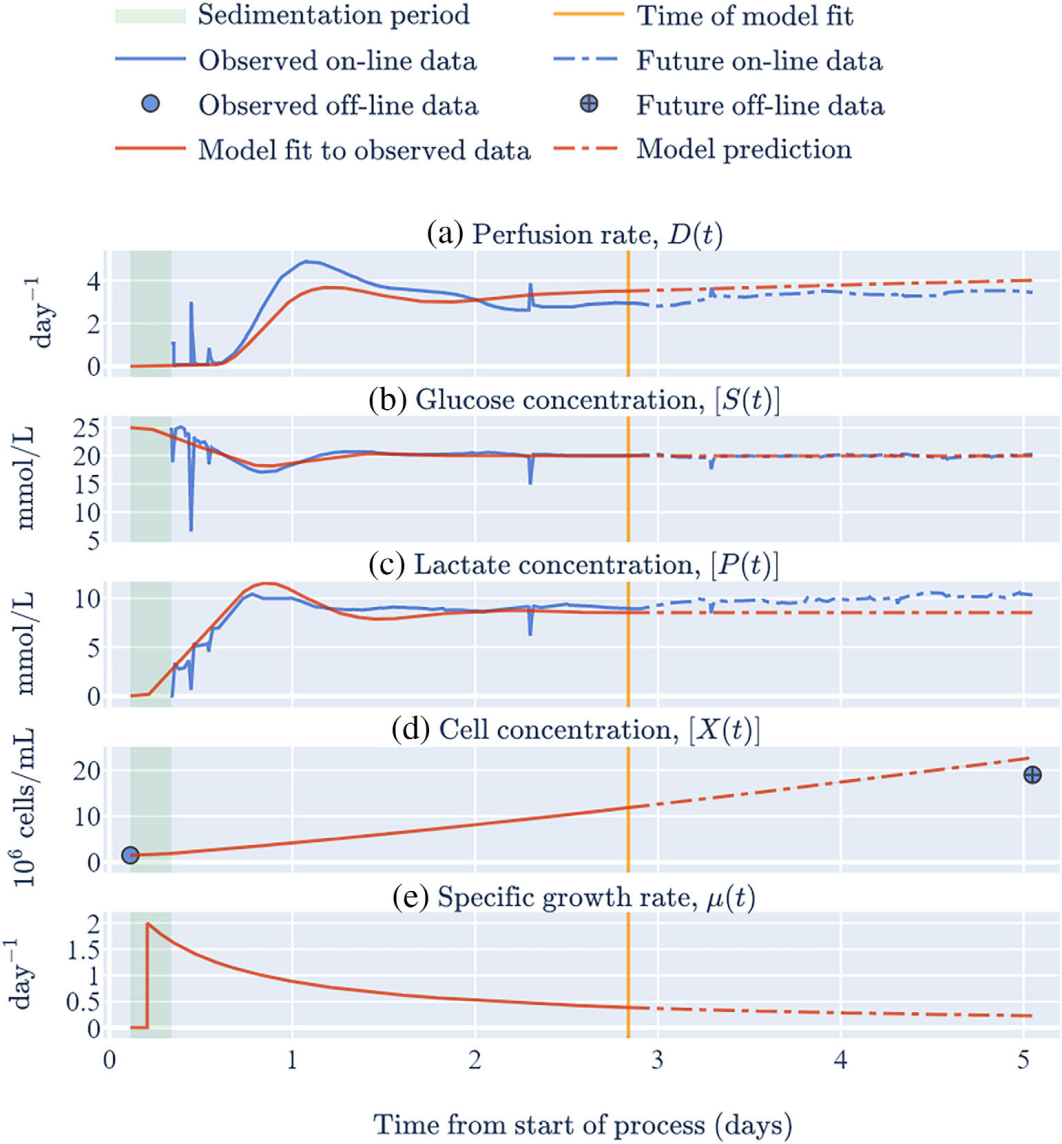
Key data from run 1 in the large chamber of the Aglaris FACER and model best‐fits to 2.5 days of online data and the off‐line initial cell concentration.

In the early process development phase of the FACER, it was found that bubbles generated during the expansion process interfered with the online sensors leading to artificial drops in the glucose/lactate concentration readings followed by large rises after the bubbles had moved away from the sensors. To prevent these fluctuations impacting the perfusion rate, the raw glucose/lactate data is filtered before being passed to the PID controller. Specifically, the filter is a Lowpass Butterworth type of order 2 with a sampling frequency of 1 Hz and a cut‐off frequency of 0.001 Hz. The glucose/lactate concentration data shown in Figures 3, 4, and S1–S9 are the filtered data. Note that not all bubble artifacts are filtered out which results in the observed ‘downward spikes’ of the glucose (and lactate) concentration followed by subsequent (of the order of seconds/minutes) ‘upward spikes’ in the perfusion rate.

Alongside the development of the glucose/lactate concentration filter, the PID parameter values ($K_{p}$, $K_{i}$ and $K_{d}$—see Table 1) were similarly determined via experimental process development. Although not yet considered optimally tuned, the PID values were found to provide satisfactory control of the glucose concentration at its set‐point. In particular, the $K_{d}$ value (i.e. the derivative gain parameter associated with the PID controller) was set 3–4 orders of magnitude lower than the $K_{p}$ and $K_{i}$ values to mitigate the impact of the bubbles on the perfusion rate.

**TABLE 1:** Observed (input) parameters of six runs in the large chamber of the Aglaris FACER. The bioreactor volume, initial cell number, and the final cell number are not explicit input model parameters but are included here for context.

Notation	Description	Units	Run 1, Donor 1, Media A	Run 2, Donor 2, Media A	Run 3, Donor 3, Media A	Run 4, Patient 1, Media A	Run 5, Donor 4, Media A	Run 6, Donor 5, Media B
$t_{I}$	Initial sedimentation time	Days	0.11	0.14	0.11	2.09	1.92	0.08
$t_{\mathrm{sed}}$	Final sedimentation time	Days	0.34	1.02	0.86	2.84	2.92	0.83
$t_{F}$	Final perfusion time	Days	5.05	6.03	6.01	7.00	6.95	4.85
$D_{\min}$	Minimum perfusion rate	Day^−1^	0.067	0.067	0.067	0.067	0.067	0.067
$k_{p}$	Proportional gain	L/(mmol*day)	0.215	0.215	0.215	0.215	0.215	0.215
$k_{i}$	Integral gain	L/(mmol*(day^2^))	5.16	5.16	5.16	5.16	5.16	5.16
$k_{d}$	Derivative gain	L/mmol	7.46 E‐05	7.46 E‐05	7.46 E‐05	7.46 E‐05	7.46 E‐05	7.46 E‐05
$\left[S_{I}\right]$	Glucose in fresh media/influent	mmol/L	25	25	25	25	25	26
$\left[S_{\mathrm{sp}}\right]$	Glucose set‐point	mmol/L	20	20	20	20	17	21
$\left[X_{I}\right]$	Initial cell concentration	10^6^ cells/mL	1.49	0.37	1.49	0.62	1.13	1.49
$\left[X_{F}\right]$	Final cell concentration	10^6^ cells/mL	19.0	19.0	32.5	10.9	8.73	18.2
V	Bioreactor volume	mL	134	134	134	134	134	134
$V\left[X_{I}\right]$	Initial cell number	10^6^ cells	200	50	200	83.1	152	200
$V\left[X_{F}\right]$	Final cell number	10^9^ cells	2.54	2.54	4.36	1.46	1.17	2.44

All datasets shown in this study are T cell or CAR T cell expansions at clinically relevant scale conducted in the FACER using variable process conditions to provide different scenarios for model training and testing. T cells were selected from peripheral blood mononuclear cells (PBMCs, Cambridge Bioscience) or leukopak (Grifols Bio Supplies) and cryopreserved. After thawing, cells were activated with TransAct™ (Miltenyi Biotec). For runs 1–5, cells were expanded in TexMACS™ medium (Miltenyi Biotec) combined with Human interleukin (IL)‐7 and Human IL‐15 (Miltenyi Biotec), henceforth referred to as “media A.” For run 6, cells were expanded in Advanced TCM, chemically defined medium (Sartorius CellGenix) with Human IL‐2 (Sartorius CellGenix) henceforth referred to as ‘media B'. Antibiotics were added in runs 1–4 and 6. Human Serum was added in runs 1–3 and 5 where donor T cells were expanded in media A but not in run 4 where patient CAR T cells (Clínica Universitaria Navarra) were expanded nor in run 6 where donor T cells were expanded in media B. Transduction in run 4 was performed with CAR lentivirus, provided by Clínica Universitaria Navarra.

### 2.3 Computational

Two programming languages were used in this study. Python code was developed for fitting the digital shadow to data from an entire historical run as described in Section 3.2 as well as fitting to data in pseudo‐real‐time as described in Section 3.3. Mathematica code was developed to perform the identifiability analysis described in section 4 of the Supporting Information.

Fitting the model to data was performed via nonlinear least‐square minimization using the Levenberg–Marquardt method in the lmfit package^44^ (referred to as the ‘fitting process’). The model was simultaneously fitted to the perfusion rate, glucose concentration, and lactate concentration data for each run. These three online data sources were given equal weight in the fitting process and no scaling was applied to any of these data. For both digital shadow training (Section 3.2) and testing (Section 3.3), Equations ((2), (3), (4))–((2), (3), (4)), (10), and (11) were used in the fitting process. In addition, for training purposes (Section 3.2), data for both the initial and final cell concentrations allowed the system to be further constrained via Equation (12), meaning that $\mu_{\max}$ did not have to be fitted directly. However, for testing purposes (Section 3.3) the unknown final cell concentration in pseudo‐real‐time meant that Equation (12) could not be used to constrain the fitting process in this scenario. An extended model that also includes Equation (8) was used to fit to the DO data, the results of which are described in Section 3.2.

Numerically solving the ODEs given by Equations ((2), (3), (4))–((2), (3), (4)) and (8) via the ‘solve.ivp’ function (within the integrate sub‐module of the SciPy package) resulted in periods of time where the solutions had negative values, particularly for the perfusion rate. Instead, these ODEs were approximated by forward finite difference equations and solved via the forward Euler method. This approach meant that the physical requirements of the system could be met by forcing the solutions to remain non‐negative at each time step. A step size of 10 s was used for two reasons; first, it is equivalent to the frequency of sensor measurements and, therefore, simplified the fitting process by allowing a direct comparison between model and online data at each step size and, second, it is small relative to the duration of the expansion periods thus resulting in a close approximation to the ODEs.

## 3 RESULTS

### 3.1 Data

Table 1 shows key time‐invariant data from six runs in the larger of the two FACER chambers. In five of these runs T cells were expanded from normal donors (runs 1–3, 5, 6) and in one run CAR T cells were expanded from a patient (run 4). The time at which the cells were seeded, $t_{I}$ is relative to the time at which data began to be collected at t=0 (i.e., the start of the process). The sedimentation period ($t_{\mathrm{sed}}-t_{I}$), was at most 1 day for all 5 runs. The range of the perfusion period ($t_{F}-t_{\mathrm{sed}}$), was ~4–5 days. This gives the range of the expansion period, denoted by $t_{F}-t_{I}$, as ~5–6 days.

The cells in runs 1, 2, 3, and 6 were seeded directly in the larger of the two chambers, whereas the cells in runs 4 and 5 had an initial expansion in the smaller chamber due to their lower initial cell number of 20 million cells (cf. the initial cell number of runs 1, 2, 3, and 6 in Table 1). The duration of this initial expansion was ~2 days, which consisted of an approximate 0.75‐day sedimentation period and 1.25‐day perfusion period prior to passage to the large chamber. The online data generated during this initial expansion period in runs 4 and 5 were not used in this paper; however, the suspension sampling conducted during passage to the large chamber allowed for a measurement of the initial cell number (in the large chamber) given in Table 1.

For training (or parameter estimation) purposes, the model was fitted to data from each of the runs 1, 2, 4, 5, and 6, thus giving donor/patient‐specific parameter values. In run 3, a mechanical issue occurred ~2.5 days following the start of perfusion and lasted for ~0.5 days. During this time, perfusion stopped, and no online data of the glucose and lactate concentrations were recorded. This loss of data makes it hard to justify using run 3 for training purposes. Instead, run 3 is used as a test dataset where only the online data prior to the mechanical issue are used, as well as the final cell concentration.

### 3.2 Digital shadow training

Figures 3 and S1–S4 show the best‐fit of the digital shadow to the data from FACER runs 1, 2, 4, 5, and 6, respectively, (i.e., to the fixed data shown in Table 1 and the time‐varying online data shown in panels a–c of each figure). Although the FACER utilizes a PID controller, the results shown here are based on a digital shadow that models a simpler PI controller because the derivative gain, $k_{d}$ was 4–5 orders of magnitude smaller than the proportional and integral gains, $k_{i}$ and $k_{p}$ for the data in this study (see Table 1). A negligible difference was found when modeling a PID controller instead of a PI controller (results not shown).

The three model parameters associated with the cell growth model can be visualized via the specific growth rate (see panel e of Figure 3) and are described as follows. After a relatively short estimated lag period ($t_{\mathrm{lag}}-t_{I}$) of approximately 4 hours (during which there is no cell growth), the cells are estimated to start growing at their maximum specific growth rate, $\mu_{\max}$, of ~2/day. The cells are then estimated to continue growing throughout the perfusion period but with a decreasing specific growth rate over time (captured by the unitless parameter ν).

Three equations provided in the Materials and Methods are consistent with three observations in Figure 3. First, substituting the decrease of the specific growth rate for run 1 (ν≈−1) into Equation (10) gives approximately linear cell growth (see panel d). Second, performing the same substitution into Equation (15) gives an approximately constant perfusion rate once the glucose concentration has stabilized at its set‐point (see panel a). Third, Equation (18) shows that regardless of the value of ν, the lactate concentration approximately stabilizes at a steady state shortly after the glucose concentration stabilizes at its set‐point (see panel c). Note that the term ‘steady state’ is used in this context rather than ‘set‐point’ because the lactate concentration is not directly controlled by the PID controller.

Table 2 shows the four fitted model parameters ($Y_{\mathrm{xs}}$, $Y_{\mathrm{px}}$, $t_{\mathrm{lag}}$, and ν) for runs 1, 2, 4, 5 and 6 as well as three values that are calculated from the fitted parameters. The parameters associated with metabolism (i.e. the two constituent yields) were the most consistent parameters across the bioreactor runs as indicated by the lowest absolute coefficients of variation (CV). The parameters associated with cell growth (i.e., the lag period, maximum specific growth rate and decrease of specific growth rate) were less consistent across the bioreactor runs than the yields (as indicated by the higher absolute CV values) suggesting that these parameters are more donor/patient‐specific. However, despite having the highest absolute CV of all the parameters, the lag period had a range of ~0–4 h. Therefore, relative to a 5–6‐day expansion period, the lag period was estimated to be of a short duration across all the runs. Indeed, in section 4.2 of the Supporting Information, it is shown that the inclusion of a lag period provides a considerable improvement in model fit (over a model without a lag period) only for run 1. It can be seen in Table S2 of the Supporting Information that including a lag period provides a marginal improvement in model fit for runs 2 and 4 and no improvement for runs 5 and 6.

**TABLE 2:** Best‐fit (output) parameters with 95% confidence intervals in parentheses for 5 runs in the large chamber of the Aglaris FACER. Note that $t_{\mathrm{lag}}$ is the parameter that is fitted but it is given here relative to $t_{I}$ to allow for an accurate calculation of the coefficient of variation (CV). $\mu_{\max}$ is not fitted directly from the data but is calculated via Equation (12) using the best‐fit parameter values of $t_{\mathrm{lag}}$ and ν. Similarly, $Y_{\mathrm{ps}}$ is calculated via Equation (5) using the best‐fit parameter values of $Y_{\mathrm{xs}}$ and $Y_{\mathrm{px}}$. In addition, $t_{T}-t_{I}$ is calculated via Equation (13) using the best‐fit parameter values of $t_{\mathrm{lag}}$ and ν, and with a threshold cell concentration of $\left[X_{T}\right]=$ 10^7^ cells/mL.

Notation	Description	Units	Run 1 Donor 1, Media A	Run 2 Donor 2, Media A	Run 4, Patient 1, Media A	Run 5 Donor 4, Media A	Run 6 Donor 5, Media B	Mean	|CV|
$Y_{\mathrm{xs}}$	Yield of cells from glucose	Cells/pmol	0.231 (0.230,0.231)	0.275 (0.275,0.276)	0.272 (0.271,0.274)	0.207 (0.207, 0.208)	0.311 (0.309, 0.313)	0.259	0.140
$Y_{\mathrm{px}}$	Yield of lactate from cells	Pmol/cell	7.93 (7.91, 7.95)	6.25 (6.23, 6.26)	9.09 (9.06, 9.13)	6.83 (6.81, 6.85)	7.99 (7.94, 8.04)	7.62	0.130
$t_{\mathrm{lag}}-t_{I}$	Lag period	Days	0.160 (0.158,0.161)	0.172 (0.164, 0.181)	0.128 (0.119, 0.137)	0.000 (0.000, 0.0188)	0.000 (0.000, 0.350)	0.092	0.831
ν	Decrease of specific growth rate	Unitless	−0.922 (−0.925, −0.918)	−0.477 (−0.480, −0.473)	−0.524 (−0.530, −0.518)	−0.000 (−0.000, −0.000)	−0.761 (−0.776, −0.747)	−0.537	0.583
$\mu_{\max}$	Max. specific growth rate	Day^−1^	2.14	2.02	1.39	0.405	1.58	1.51	0.409
$Y_{\mathrm{ps}}$	Yield of lactate from glucose	Unitless	1.83	1.72	2.48	1.42	2.49	1.99	0.215
$t_{T}-t_{I}$	Required expansion period	Days	2.59	4.14	4.65	5.38	2.71		
$\chi_{n}^{2}$	Reduced chi‐squared	Unitless	0.604	0.578	0.753	0.807	3.17		

In addition, Table 2 shows that the decrease of the specific growth rate for runs 2 and 4 (ν≈−1/2) was approximately half that obtained for run 1. Substituting this value into Equations (10) and (15), respectively, gives a cell concentration that is predicted to increase quadratically with time and a perfusion rate that is predicted to increase linearly with time (once the glucose concentration has stabilized at its set‐point). These features are consistent with the model fits shown in Figures S1 and S2. This contrasts with run 1 where the cell concentration is predicted to increase linearly, and the perfusion rate is predicted to remain constant after the initial transient phase.

The yield of lactate from glucose, $Y_{\mathrm{ps}}$ is given by the product of the constituent fitted yields (see Equation (5) in the Materials and Methods). Table 2 shows that $Y_{\mathrm{ps}}$, with a mean of 1.99 (unitless), is consistent with the aerobic glycolysis metabolic pathway where 2 mols of lactate is produced for every mol of glucose consumed.^45^, ^46^, ^47^, ^48^ Table 2 also includes a prediction of the expansion period required to reach a threshold cell concentration of 10^7^ cells/mL (i.e. 10 million cells per mL) using Equation (13) in the Materials and Methods and the fitted model parameter values. These periods range from ~2.5 days for runs 1 and 6 to ~5.5 days for run 5. This 3 day difference in the predicted required expansion period is partly due to the initial cell concentration being 1/3 higher in runs 1 and 6 than in run 5 (see Table 1) but also due to the cells growing faster in the early stages of runs 1 and 6 than in run 5 (compare the maximum specific growth rate of runs 1, 5, and 6 in Table 2).

A visual comparison of model and data in Figures 3 and S1–S4 shows a reasonable‐to‐good model fit. This is supported by the reduced chi‐squared statistic, $\chi_{n}^{2}$, with values in Table 2 ranging from 0.58 to 0.81 for four of the training runs. For run 6, $\chi_{n}^{2}\approx 3.2$, which is likely a consequence of oscillations in the data, possibly induced by the choice of PID parameters (this point is addressed further in the Discussion). Although $\chi_{n}^{2}<1$ can indicate that the model is overfitting the data, this is very unlikely to be the case here because it is shown in section 4.2 of the Supporting Information and Table S1 that the model is structurally identifiable.^49^, ^50^, ^51^, ^52^ This means that it is possible to identify the unknown model parameters under the assumption of noise‐free, continuous observations of the system. Such structural identifiability provides greater confidence that the fitted parameters shown in Table 2 are meaningful, and not simply one set of multiple parameter combinations that could provide equally good fits to the data. To verify this further, Table S3 shows two goodness‐of‐fit measures, $\chi_{n}^{2}$ and Akaike's Information Criterion (AIC), for a range of initial parameter values. For most initial values, the fitting process converges to the best‐fit parameters shown in Table 2. For some initial values, the fitting process results in an alternative model fit but in all such cases both the $\chi_{n}^{2}$ and AIC values were larger than those shown in Table 2. Such larger values suggest an inferior model fit to the original fit, and this was confirmed in all cases via visual inspection (results not shown). Collectively, this indicates that the best‐fit values shown in Table 2 are the global minimum, but that the choice of initial values is important to avoid a model fit that is associated with a local minimum.

The ODE for the DO concentration given by Equation (8) has a similar form to the ODE for the glucose concentration given by Equation (3). A key difference is that Equation (8) includes the volumetric mass transfer coefficient, $k_{L}a$. This means that extending the digital shadow via the addition of Equation (8) requires the estimation of two additional parameters (i.e., $k_{L}a$ and the (scaled) yield of cells from DO, $Y\prime_{\mathrm{xc}}$) via fitting to the DO data (while still simultaneously fitting to the perfusion rate and glucose/lactate concentration data). Table S4 shows that both fitted parameters have very wide confidence intervals relative to those in Table 2. This suggests that $k_{L}a$ and $Y\prime_{\mathrm{xc}}$ are not practically identifiable parameters, that is, that the parameter estimates obtained via fitting to the DO data are unreliable in this context. As such, given the absence of prior knowledge of $k_{L}a$ (or $Y\prime_{\mathrm{xc}}$) for the data in this study, more robust predictions of cell concentration are achieved via the digital shadow that utilizes the perfusion rate and glucose/lactate concentrations, but that does not additionally utilize the DO data. However, despite the highly uncertain parameter estimates, Figures S5‐S9 show that it is possible to get reasonable fits to the DO data (while still fitting the other online data) via the addition of a further simple ODE given by Equation (8).

The fitting method described in the Materials and Methods allows for the calculation of confidence intervals of the best‐fit parameter values. It can be seen in Table 2 that the confidence intervals are narrow for all fitted parameters. This is likely a result of the digital shadow consisting of only four fitted parameters while being constrained to simultaneously fit three sets of online data as well as the initial and final cell concentration data. Consequently, confidence bands around the cell concentration predictions in Figure 3 are indistinguishable from the best‐fit values.

### 3.3 Digital shadow testing

It is shown in section 4.2 of the Supporting Information and Table S1 that in a real‐time scenario, either the lag period or the maximum specific growth rate must be known a priori for the model to be structurally identifiable. As such, for the real‐time analysis that follows below, the lag period is no longer a parameter to be fitted but instead is fixed at its mean value from Table 2. The reason for fixing the lag period rather than the maximum growth rate is that the lag period was consistently of the order of just a few hours in Table 2, which is short relative to the expansion period.

It was found that fixing the lag period alone led to poor predictions of the final cell concentration, even if all the online data were available (results not shown). This suggested that although the model is structurally identifiable when the lag period is known a priori, the model is not practically identifiable (i.e., some or all of the fitted parameter values are highly uncertain). To resolve this issue, in addition to having a fixed lag period, the yield of cells from glucose was also fixed at its mean value from Table 2 because this parameter was consistent across the bioreactor runs (as indicated by a low CV). Furthermore, this parameter would always be required in the digital shadow (providing that the PID controller acted to control the glucose concentration) unlike the yield of lactate from cells which could potentially be removed from the digital shadow in the absence of a lactate sensor. This meant that in the real‐time version of the model, two parameters ($t_{\mathrm{lag}}$ and $Y_{\mathrm{xs}}$) were fixed and the remaining three parameters ($Y_{\mathrm{px}}$, $\mu_{\max}$, and ν) were fitted to the incoming data. Figure 4 shows the model fit for run 1 with 2.5 days of online data (i.e., data from the first 2.5 days of the perfusion period), indicating that it is possible to get relatively accurate cell concentration predictions in real‐time via this approach.

**FIGURE 5:**
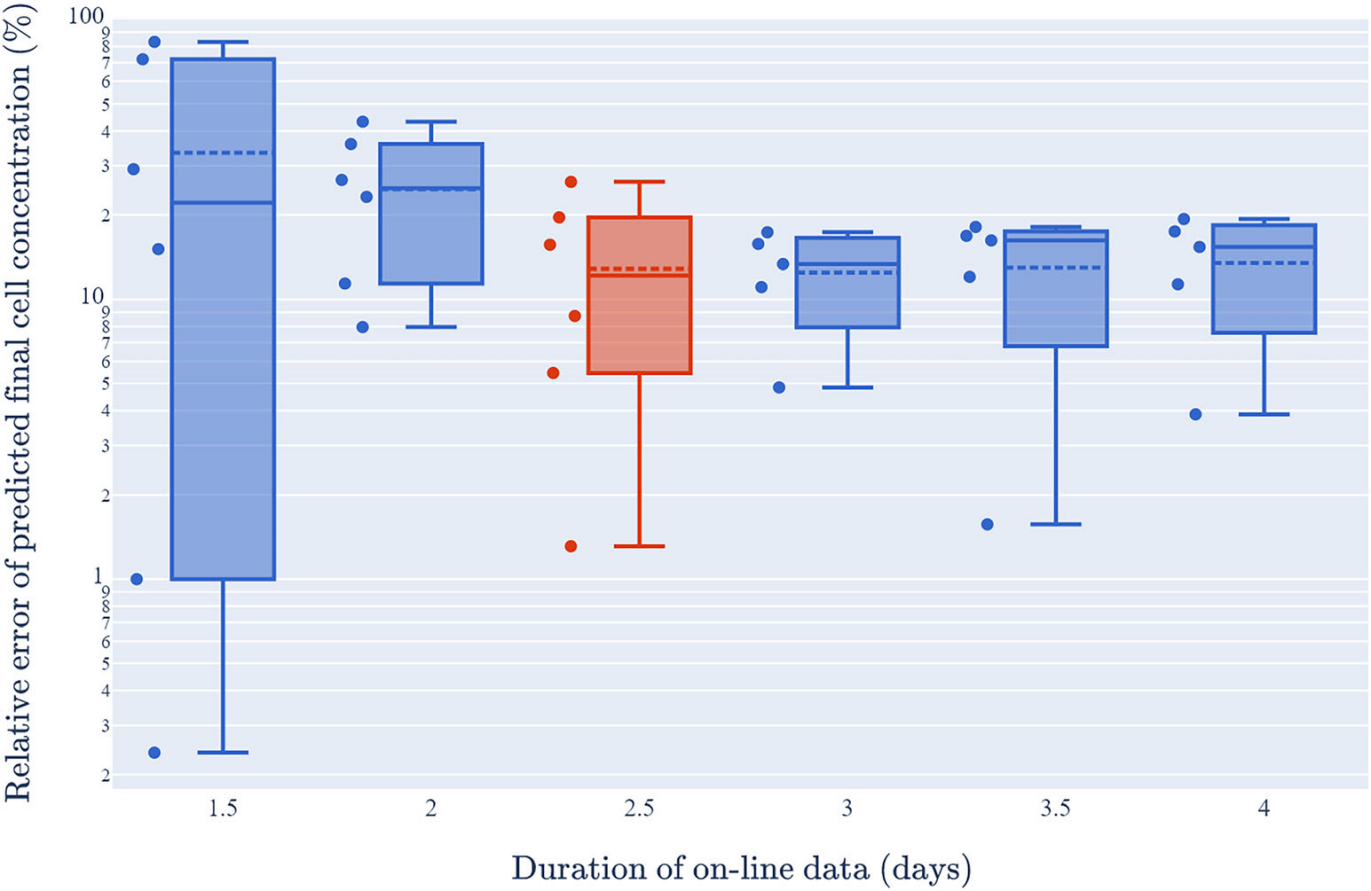
Boxplots of relative errors of predicted final cell concentration with varying quantities of online data. Relative errors for runs 1, 2, 4, 5, and 6 shown with 1.5–4 days of online data. Relative errors for run 3 shown with 1.5–2.5 days of online data (due to the mechanical issue). Within each box, the solid horizontal line represents the median and the dotted horizontal line represents the mean. The red boxplot indicates predicted cell concentrations shown in Figure 6.

Due to the limited number of runs that were available for both training and testing purposes, the real‐time version of the model was fitted to each of the training runs but with only 0.5 to 4 days of online data (in half‐day time steps) rather than with complete online data. Testing with such incomplete training data, especially when combined with fixing only 2 of the 5 required parameters at their averaged trained values, provides a justifiable validation of the digital shadow. In addition, the real‐time model was fitted to online data for run 3 (0.5 to 2.5 days) which is the independent test run that had not been used as a training dataset in Table 2. Figure 5 shows that with 1.5 days of online data, although for four of the runs the model predicted the final cell concentration to within a 30% relative error, for two of the runs the model gave a relative error of over 70%. However, with 2.5 days (or more) of online data, the model predicted the final cell concentration to within a 30% relative error for all the runs, with a mean relative error of 13% (standard deviation ≈ 6%). This suggests that 2.5 days of online data is required before the model can consistently provide reasonable‐to‐good cell concentration predictions.

**FIGURE 6:**
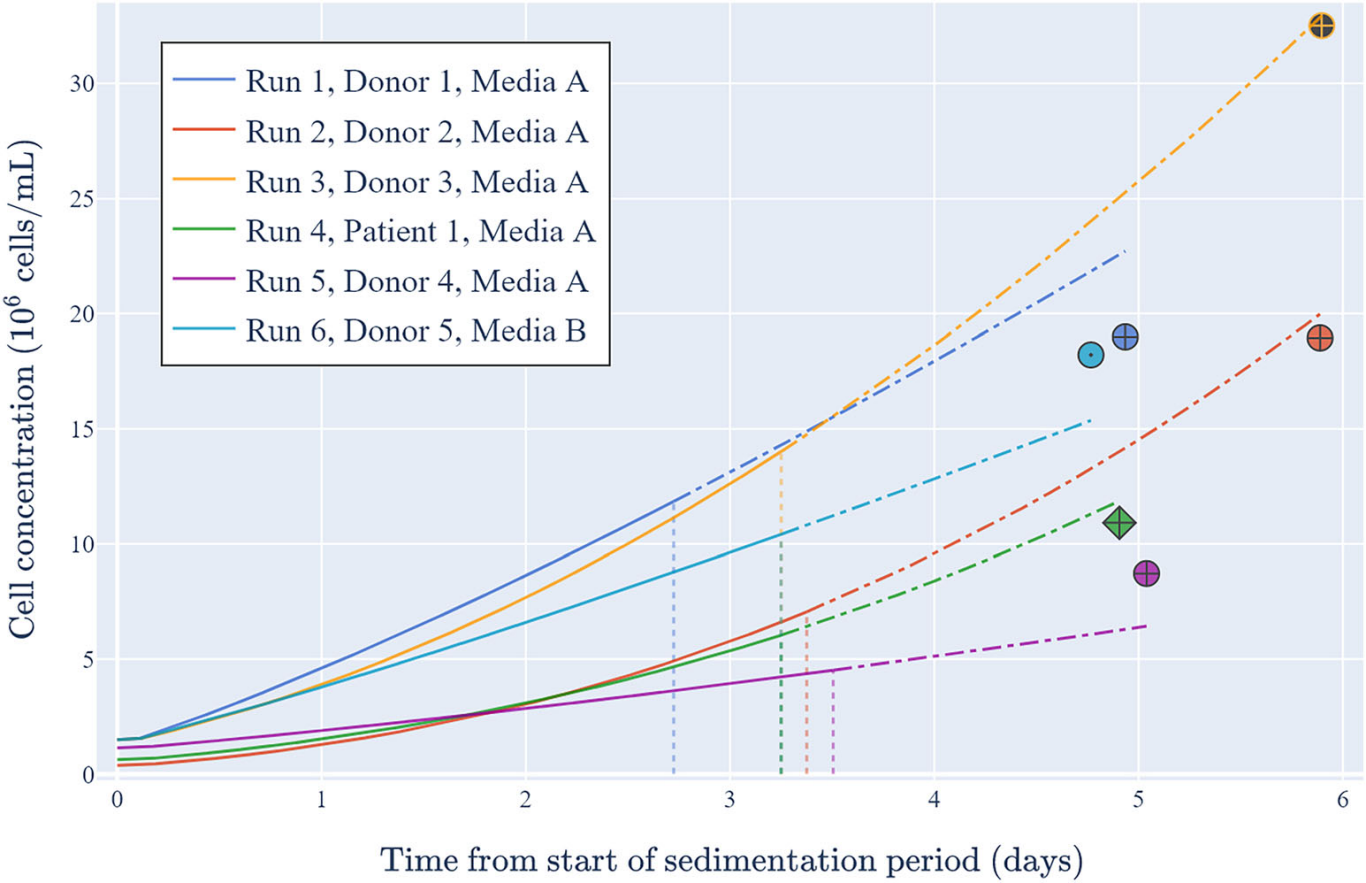
Cell concentration predictions based on 2.5 days of online data the off‐line initial cell concentration. Full lines represent predictions in the past and up until the present time that the model is fit. Dash‐dotted lines represent future predictions. Dotted vertical lines represent the time that the model is fit. Symbols represent the off‐line final cell concentrations in which circles represent donors, the diamond represents patient 1, colored filled symbols represent training runs, the black filled symbol represents the independent test run, crosses represent media A and the dot represents media B.

Equation (10) in the Materials and Methods gives the novel model of early cell growth that is utilized in the digital shadow. Equation (10) is used in Figure 6 to show the cell concentration predictions based on 2.5 days of online data for the six bioreactor runs (the predictions with 3, 3.5, and 4 days of online data are broadly similar as per Figure 5). Note that this was the final time at which run 3 had complete online data prior to the loss of data for approximately half a day. It is shown that within 3.5 days of the start of expansion, the real‐time model captures the donor/patient variability in cell growth and provides reasonable (and sometimes very good) predictions of cell concentrations 1.5–2.5 days into the future. For the five training runs, the model does not consistently under‐ or over‐predict the final cell concentration. With test run 3, the model correctly predicts higher cell concentrations to those observed in the training runs (i.e., predictions outside the training domain), arguably providing further model validation.

In addition, the derivation of confidence bands for the cell concentration and specific growth rate predictions are provided in section 3.10 of the Supporting Information. It can be seen in the Videos [Link], [Link] that the confidence bands narrow as more online data becomes available. The confidence bands for all runs are too narrow in that the final cell concentration is frequently not within the band. This is probably because there are other sources of uncertainty that are not captured by this approach, for example, uncertainty in $Y_{\mathrm{xs}}$ and $t_{\mathrm{lag}}$, uncertainty in the online and off‐line measurements, and uncertainty in the model structure. As such, the confidence bands shown in the Videos [Link], [Link] should be interpreted as *minimum* confidence bands. Despite the challenges of capturing all the uncertainty, these results show that with at least 2.5 days of online data, the digital shadow generally predicts the cell concentration (up to 2.5 days into the future) with a relative error of less than 20%.

## 4 DISCUSSION

An important feature of this paper is the application of a novel model of cell growth in which the specific growth rate can decrease over time to reflect the cells competing for space to grow in an increasingly crowded environment. Specifically, we have derived a special case of the generalized logistic growth model first described by Richards.^43^, ^53^ This special case is applicable in the early stages of cell growth when cells are expanding rapidly and yet still much lower in concentration than the carrying capacity (or the maximum possible cell concentration). The special case consists of only two parameters—the first is the initial (or maximum) specific growth rate, $\mu_{\max}$ and the second parameter, ν, dictates the decrease in the specific growth rate over time. The simplicity of this model makes it appealing because it allows for a range of cell growth patterns including linear and quadratic via a relatively straightforward equation. Although not strictly applicable to the conditions of the special case, increasingly small values of ν recover the limiting case of exponential growth (i.e., an effectively constant specific growth rate). This also happens to be a special case of the standard logistic growth model, first described by Verhulst,^54^ in the early stages of cell growth. However, for five of the six bioreactor runs analyzed in this paper, the additional complexity of a decreasing, rather than constant, specific growth rate over time has provided a sufficiently improved fit to the data. The simple functional form of our cell growth model also allows for further insight into the bioreactor process described here. For example, it has been shown that providing the glucose concentration has stabilized at its set‐point, a predicted linear/quadratic cell growth profile results in an approximately constant/linearly increasing perfusion rate.

Utilizing the special case rather than the full Richards cell growth model can also be justified with the following reasoning. First, the carrying capacity of a given bioreactor may be fixed but uncertain or may vary with donor/patient. Second, although the digital shadow that includes the full Richards model was shown to be structurally identifiable, fitting this model to the bioreactor data often resulted in large confidence intervals associated with the carrying capacity estimates (see section 4.2 of the Supporting Information for details). This suggested that the carrying capacity was not a practically identifiable parameter (i.e., that there was considerable uncertainty associated with the fitted values of the carrying capacity) given the available data. In addition, the AIC values in Table S2 are often similar, suggesting that the special case was just as good as the full Richards model at explaining the data. Third, the assumed constant constituent yields were found to be relatively consistent across the five training runs and the product of these yields (i.e., the yield of lactate from glucose) was generally found to be consistent with aerobic glycolysis where 1 mole of glucose ultimately gets converted to 2 moles of lactate.^27^, ^45^, ^46^, ^47^, ^48^ It has been shown that this metabolic pathway is associated with early cell growth and that a switch in metabolism generally occurs once the cells enter the stationary phase,^15^, ^22^, ^55^ for example, when the cell concentration approaches the carrying capacity. Thus, the simplifying assumptions of time‐invariant yields and a cell growth model applicable at early times in the expansion period are consistent with each other and consistent with the bioreactor data presented in this paper.

Although the digital shadow would ideally be trained and tested on more than six bioreactor runs, we have taken a pragmatic approach to the experimental validation of the model. The inherent complexity of working with primary cells from donors and patients (along with the high costs and the time‐intensive nature of preparing and analyzing each run for cell therapy applications) has limited the number of experiments. On the other hand, the number of runs currently available supports the development of a digital shadow, rather than a digital twin, because the latter would require many more datasets to validate, especially if it were seeking to replace PID control with, for example, model‐based control strategies.^41^ A further benefit of a digital shadow is that it requires less regulatory oversight than a digital twin, because the former has no direct control of the process but is instead an advisory tool for a human operator who ultimately oversees the process. Indeed, in relation to the recent European Union's Artificial Intelligence (AI) Act,^56^ we would contend that the digital shadow presented here could be considered minimal‐to‐low risk when viewed as an AI system because it “is intended to perform a narrow procedural task” (that of predicting the cell concentration in real‐time) and “is intended to improve the result of a previously completed human activity” (that of deciding the time at which to harvest the cells).^57^

Additional bioreactor runs would also help to better understand whether the inclusion of the lag period at the start of the expansion period is warranted. For example, in this study only one of the training runs showed a considerable improvement in model fit with the inclusion of a lag period, two of the training runs showed a marginal improvement, and two training runs showed no improvement (associated with a predicted lag period of zero days duration). Given that, at most, the predicted lag period was of the order of a few hours, this supported our justification for fixing this parameter in the real‐time version of the model. Indeed, using the version of the model without a lag period would likely have resulted in similar real‐time predictions to those shown in the Results. However, the method of inserting the CAR gene into the T cells could potentially have a considerable impact on the lag period. Although transducing the CAR gene via the lentiviral approach does not appear to have had an impact in run 4, transfection via electroporation could potentially result in a delay of the order of days, rather than hours, before the cells start to grow. Given that the structural identifiability analysis performed here has shown that either the lag period or the maximum specific growth rate is required to be known when implementing the real‐time version, any future operational use of the digital shadow that includes electroporation would ideally have first been re‐trained on new bioreactor runs that utilize this method. Partly for this reason, we felt it was important to include in this paper the model with a lag period despite the short periods predicted for the five training runs. It is also worth emphasizing that CAR expression levels resulting from electroporation (and other techniques) can be considerably less than 100%,^58^ and in such cases it would be more informative for the digital shadow to provide predictions of T cells with CARs expressed on their surface rather than all T cells. Again, future bioreactor runs could help to characterize the CAR expression profile during expansion via sampling of sedimented (rather than suspended) cells. Such insights could then be incorporated into updated versions of the digital shadow described in this paper.

A further improvement to the digital shadow would be to provide confidence bands that capture the uncertainty in the yield of cells from glucose and the lag period; two parameters that are held constant when operating in real‐time. Additional bioreactor runs would allow for an improved quantitative understanding of the bivariate distribution of these parameters, and the extent to which they are correlated. For example, having fitted a bivariate distribution to the parameter estimates of $Y_{\mathrm{xs}}$ and $t_{\mathrm{lag}}$ from many runs, it would then be possible to repeatedly sample from such a distribution, and use such sampled values when fitting to the online data in real‐time. This would provide a distribution of predicted cell concentrations that would provide more realistic confidence bands to those shown in Videos [Link], [Link].

Our results show that an extended digital shadow that additionally fits Equation (8) to DO data would require prior knowledge of the volumetric mass transfer coefficient (i.e. the $k_{L}a$ parameter) so that the remaining parameter values could be reliably estimated. One approach to estimate $k_{L}a$ is the non‐steady state method^34^ (also known as the dynamic gassing out method^42^, ^59^) in which the bioreactor (without any cells present) is deoxygenated by passing, for example, nitrogen through the vessel, and then the DO concentration is measured once a standard composition of gas is subsequently provided. The resulting DO data could then be fitted by Equation (8) (in the absence of the OUR term) to give an estimate of $k_{L}a$ for that combination of bioreactor/media/gas composition, and so forth.

Perhaps the strongest assumption in our model is that the media and cells are homogeneously mixed. Indeed, in the physical bioreactor it is desirable for the cells to sediment on the gas‐permeable membrane, and for the media to enter/exit toward the top to prevent cells from being removed in the effluent. As the cells continue to proliferate and begin to “stack” upon each other it is possible that cells toward the bottom receive more oxygen from below and less glucose from above than cells toward the top (and vice versa). Furthermore, the perfusion of media may result in laminar flow between the influent and effluent with limited lateral mixing, again resulting in regions with variable quantities of fresh and spent media. In addition to the assumption of homogeneous mixing there is also an implicit assumption that all the cells in each bioreactor run are homogeneous in that they all metabolize glucose at the same constant rate and grow at the same time‐dependent rate. However, T cells are generally composed of cells that express either CD4 or CD8 receptors on their surface and these subsets have been shown to have different expansion profiles.^17^, ^60^ Further research to better understand any considerable differences between these (or other) subsets would allow the current unsegregated model to be developed into an even more patient‐specific segregated model.^41^ Such an enhanced model could also be used to guide the initial composition (i.e. ratio) of CD4+ to CD8+ T cells seeded into the bioreactor because it has been shown that the final ratio can impact the overall effectiveness of CAR T cells.^60^ In addition, extending the current deterministic model into a stochastic model that can capture fluctuations in cell metabolism/growth and/or sensor measurements may help to explain the oscillations sometimes observed in the online data (see Figures S1–S4). However, in this study we have shown that a simple deterministic model based on the assumption of homogeneity within the bioreactor can explain the online and off‐line data of the six bioreactor runs reasonably well (and very well in some cases).

## 5 CONCLUSION

We have developed a digital shadow of cell growth in a perfusion bioreactor where a PID controller acts to maintain the glucose concentration at its set‐point by frequently adjusting the perfusion rate. One benefit of this digital shadow is that it does not require any additional PAT, such as a bio‐capacitance probe, to be incorporated into the bioreactor because it utilizes the hard sensors that are already being used to monitor and control the process. A second benefit is that the digital shadow could be used in the future to tune the parameters of the PID controller whose current values have been identified through experimental trial and error. This may help to improve the stability of the glucose concentration around its set‐point, which could be advantageous to cell growth as well as improving the predictive capability of the digital shadow during a bioreactor run. A third benefit is that the digital shadow could be considered minimal‐to‐low risk from a regulatory perspective because it performs a narrow task that provides advice to a human operator overseeing the process. Indeed, the fourth and main benefit is that the digital shadow can provide nowcasts and short‐term (i.e., a few days) forecasts of the CAR T cell concentration in real‐time. As such, this paper presents a novel soft sensor of cell concentration in a metabolite‐controlled perfusion bioreactor. The soft sensor is particularly beneficial for bioreactors where cell retention occurs via sedimentation because of the challenge of utilizing hard sensors in such an environment combined with the disruption of resuspending the cells to take samples. The ability to predict cell concentration can inform decision‐making regarding the harvest time of the CAR T cells as well as production scheduling of the manufacturing platform. Ultimately, this could help to administer each patient with a product of optimal quantity and quality as well as helping to maximize the number of patients receiving CAR T cell therapy.

## AUTHOR CONTRIBUTIONS

**Joseph R. Egan:** Conceptualization; data curation; formal analysis; investigation; methodology; project administration; software; validation; visualization; writing—original draft. **Núria Marí‐Buyé:** Conceptualization; data curation; funding acquisition; investigation; methodology; project administration; resources; validation; writing—original draft. **Elia Vallejo Benítez‐Cano:** Data curation; investigation; methodology; validation; writing—review and editing. **Miquel Costa**: Data curation; funding acquisition; investigation; methodology; resources; supervision; validation; writing—review and editing. **Linda Wanika:** Formal analysis; investigation; methodology; software; visualization; writing—original draft. **Michael J. Chappell:** Funding acquisition; methodology; project administration; resources; supervision; validation; writing—review and editing. **Ursula Schultz:** Data curation; investigation; validation; writing—review and editing. **Jelena Ochs:** Data curation; investigation; validation; writing—review and editing. **Manuel Effenberger:** Data curation; investigation; validation; writing—review and editing. **David Horna:** Conceptualization; data curation; funding acquisition; methodology; resources; supervision; validation; writing—review and editing. **Qasim Rafiq:** Conceptualization; funding acquisition; project administration; supervision; writing—review and editing. **Stephen Goldrick:** Conceptualization; data curation; funding acquisition; methodology; project administration; resources; supervision; validation; visualization; writing—review and editing.

## FUNDING INFORMATION

This project has received funding from the European Union's Horizon 2020 research and innovation program under grant agreement No. 101016909. The materials presented and views expressed here are the responsibility of the authors(s) only. The EU Commission takes no responsibility for any use made of the information set out. Funding from the UK Engineering & Physical Sciences Research Council (EPSRC) for the ‘Future Targeted Healthcare Manufacturing Hub' and the ‘FAST CAR‐T: Faster, Adaptive and Scalable Technologies for CAR‐T Manufacture’ grants hosted at University College London with UK project partners is gratefully acknowledged (Grant References: EP/P006485/1 and EP/Z532770/1, respectively). The production of some of the datasets used in the model were funded by the Spanish Ministerio de Economía y Competitividad (Retos Colaboración RTC‐2017‐6578‐1) and Innovate UK (Project 830,121), granted to Aglaris Cell S.L. and Aglaris Ltd., respectively.

## CONFLICT OF INTEREST STATEMENT

The authors NMB, EV, MC, and DH are employed by Aglaris Cell. Authors EV, MC, and DH are employed by Aglaris Ltd. Authors US, JO, and ME are employed by Sartorius CellGenix GmbH. The remaining authors declare that the research was conducted in the absence of any commercial or financial relationships that could be construed as a potential conflict of interest.

## Supporting information


**Data S1.** Supporting Information.

**Supplementary Video S1.** Key data from run 1 in the large chamber of the Aglaris FACER and model best‐fits to increasing durations of online data and the off‐line initial cell concentration.

**Supplementary Video S2.** Key data from run 2 in the large chamber of the Aglaris FACER and model best‐fits to increasing durations of online data and the off‐line initial cell concentration.

**Supplementary Video S3.** Key data from run 3 in the large chamber of the Aglaris FACER and model best‐fits to increasing durations of online data and the off‐line initial cell concentration.

**Supplementary Video S4.** Key data from run 4 in the large chamber of the Aglaris FACER and model best‐fits to increasing durations of online data and the off‐line initial cell concentration.

**Supplementary Video S5.** Key data from run 5 in the large chamber of the Aglaris FACER and model best‐fits to increasing durations of online data and the off‐line initial cell concentration.

**Supplementary Video S6.** Key data from run 6 in the large chamber of the Aglaris FACER and model best‐fits to increasing durations of online data and the off‐line initial cell concentration.

## Data Availability

The datasets presented in this article are not readily available to protect proprietary information. Requests to access the datasets (specifically the online perfusion rates, the glucose and lactate concentrations, and their associated time points of the six bioreactor runs) should be directed to Núria Marí Buyé (nuria@aglariscell.es).
