# Supplementary material for: A digital shadow of CAR T cell expansion in a perfusion bioreactor: Informing optimal harvest times for autologous cell therapy
Source: Biotechnol Prog. 2025 Jun 23;41(6):e70045. doi: 10.1002/btpr.70045 (PMC12696450; doi:10.1002/btpr.70045)
Supplement: Supplementary file 1 — Data S1. Supporting Information. [file BTPR-41-e70045-s001.pdf]

Supplementary Material

This Supplementary Material consists of six sections. The mechanistic mathematical digital shadows described in sections 1-3 are, henceforth, collectively referred to as the 'digital shadow'. Specifically, in section 1 a model describing a perfusion bioreactor that includes an initial batch period is described. In section 2 a proportional-integral-derivative (PID) control model is described along with the numerical solution that is used to fit the digital shadow to the bioreactor data. In section 3 eight models of cell growth are described, four of which are approximations to the other four exact versions. In section 4 a description of the identifiability analysis performed on the digital shadow is provided along with a description of the goodness-of fit-metrics for the key models. Finally, supplementary tables are shown in section 5 and supplementary figures are shown in section 6.

1 BATCH-PERFUSION PROCESS

Let $[X(t)] > 0$, $[S(t)] \geq 0$, and $[P(t)] \geq 0$ denote the concentration of cells, glucose and lactate, respectively. Also let $V(t) > 0$ denote the volume of the bioreactor and $Q(t) \geq 0$ denote the volumetric flow rate with dimensions of volume/time. Consider the following process. The influent flow rate is equal to the effluent flow rate which means that $V(t) = V$ (i.e. the volume of the bioreactor remains constant). The influent contains only fresh media (i.e. no cells nor lactate) with glucose concentration denoted by $[S_{\mathrm{I}}]$. The cells are seeded into fresh media within the bioreactor at time t_{I} with initial conditions given by $[X(t_{\mathrm{I}})] = [X_{\mathrm{I}}]$, $[S(t_{\mathrm{I}})] = [S_{\mathrm{I}}]$ and $[P(t_{\mathrm{I}})] = [P_{\mathrm{I}}] = 0$. Perfusion does not begin immediately at time t_{I} but at some later time t_{sed}. The volumetric flow rate is therefore given by:

$$Q(t) = 0 \quad \text{for } t_{\mathrm{I}} \leq t \leq t_{\mathrm{sed}}. \tag{S1}$$

Equation S1 means that the bioreactor undergoes an initial batch process prior to a subsequent perfusion process, henceforth referred to as a 'batch-perfusion process'. Further consider that during the batch process, cells sediment at the bottom of the vessel. As such, the period in which the batch process occurs is henceforth referred to as the 'sedimentation period'. Subsequent perfusion takes place during the 'perfusion period'. In addition to the process described thus far, the digital shadow outlined below is based on the following six key assumptions:

- The volumetric flow rate is sufficiently small that cells remain sedimented at the bottom of the vessel and only a negligible number of cells are removed in the effluent.
- Despite cell sedimentation at the bottom and the influent/effluent being situated higher up, the vessel is sufficiently shallow that the bioreactor can be considered homogeneous (i.e. the cells, fresh media and spent media are well mixed).
- Via a combination of a sufficiently short sedimentation period and subsequent perfusion, the glucose concentration remains sufficiently high (i.e. much greater than the 'saturation constant'), and the lactate concentration remains sufficiently low (i.e. much lower than the 'inhibition constant'), that both concentrations have only a negligible inhibitory effect on cell growth.
- Thus, instead of substrate/product inhibition, the dominant inhibitory effect on cell growth is due to the cells themselves competing with each other to grow in a space-limited environment.
- The rate of cell death, and the rates of glucose/lactate consumption/production associated with cell maintenance are negligible compared to those rates associated with cell growth.
- The growth yield factor of cells from glucose and growth yield factor of lactate from cells are constant.

With these assumptions, a mass balance on the cells, glucose and lactate concentrations in the bioreactor leads to the following ordinary differential equations (ODEs):

$$\frac{d[X]}{dt} = \mu(t)[X(t)] \quad \text{for } t \geq t_{\mathrm{I}}, \tag{S2}$$

$$\frac{d[S]}{dt} = H\left(t - t_{\mathrm{sed}}\right) D(t) \left([S_{\mathrm{I}}] - [S(t)]\right) - \frac{1}{Y_{\mathrm{xs}}} \frac{d[X]}{dt} \quad \text{for } t \geq t_{\mathrm{I}}, \tag{S3}$$

$$\frac{d[P]}{dt} = Y_{\mathrm{px}} \frac{d[X]}{dt} - H\left(t - t_{\mathrm{sed}}\right) D(t)[P(t)] \quad \text{for } t \geq t_{\mathrm{I}}. \tag{S4}$$

In equation S2, $\mu(t)$ is the specific growth rate as given by Richards' model of cell growth [1] and is considered further in section 3. In equations S3 and S4, $H(t)$ denotes the Heaviside step function which is used to capture the batch process followed by the perfusion process, and $D(t)$ is the dilution rate (henceforth referred to as the 'perfusion rate') given by:

$$D(t) = Q(t)/V \quad \text{for } t \geq t_{\mathrm{I}}. \tag{S5}$$

Both $\mu(t)$ and $D(t)$ have dimensions of time^{-1}. Also in equations S3 and S4, the parameters Y_{xs} and Y_{px} denote the growth yield factor of cells from glucose, and the growth yield factor of lactate from cells, respectively [2, p. 576]. Note that the growth yield factor of lactate from glucose, Y_{ps} is given by the product of the two constituent growth yield factors:

$$Y_{\mathrm{ps}} = Y_{\mathrm{px}} Y_{\mathrm{xs}}. \tag{S6}$$

Equations S3 and S4 can be integrated to give the following equations:

$$[S(t)] = [S_{\mathrm{I}}] - \frac{[X(t)] - [X_{\mathrm{I}}]}{Y_{\mathrm{xs}}} \quad \text{for } t_{\mathrm{I}} \leq t \leq t_{\mathrm{sed}}, \tag{S7}$$

$$[P(t)] = Y_{\mathrm{px}} \left([X(t)] - [X_{\mathrm{I}}]\right) \quad \text{for } t_{\mathrm{I}} \leq t \leq t_{\mathrm{sed}}. \tag{S8}$$

Note that equations S7 and S8 are applicable during the initial sedimentation period. Combining equations S1 and S5 gives the perfusion rate in the sedimentation period:

$$D(t) = 0 \quad \text{for } t_{\mathrm{I}} \leq t \leq t_{\mathrm{sed}}. \tag{S9}$$

In section 3 it is shown that analytical solutions exist for the cell concentration, $[X(t)]$. Combining such solutions with equations S7, S8 and S9 provide complete analytical solutions for the sedimentation period. Furthermore, utilizing the analytical solutions of $[X(t)]$ and $\mu(t)$ described in section 3, the set of ODEs given by equations S2, S3 and S4 reduce to the following ODEs:

$$\frac{d[S]}{dt} = D(t)\left([S_\mathrm{I}] - [S(t)]\right) - \frac{\mu(t)[X(t)]}{Y_\mathrm{xs}} \quad \text{for } t_\mathrm{sed} < t, \tag{S10}$$

$$\frac{d[P]}{dt} = Y_\mathrm{px}\mu(t)[X(t)] - D(t)[P(t)] \quad \text{for } t_\mathrm{sed} < t. \tag{S11}$$

Equations S10 and S11 provide two of the three equations required to determine the system during the perfusion period. The remaining ODE of the perfusion rate is described in section 2 below.

2 PROCESS CONTROL

Let us consider the scenario where, once perfusion begins, the perfusion rate is not set at a constant value but instead can vary with time. Furthermore, if the perfusion rate varies via a PID controller that acts to maintain the glucose concentration at a constant set-point, $[S_\mathrm{sp}]$, then the equation describing the PID controlled perfusion rate is given by:

$$D(t) = D(t_\mathrm{sed}) + K_\mathrm{p}\big([S_\mathrm{sp}] - [S(t)]\big) + K_\mathrm{i}\int_{t_\mathrm{sed}}^{t}\big([S_\mathrm{sp}] - [S(t)]\big)\,d\tau + K_\mathrm{d}\frac{d}{dt}\big([S_\mathrm{sp}] - [S(t)]\big) \quad \text{for } t_\mathrm{sed} < t, \tag{S12}$$

where $K_\mathrm{p} > 0$, $K_\mathrm{i} > 0$ and $K_\mathrm{d} > 0$ denote the constant coefficients of the proportional, integral and derivative terms, respectively. Assuming that $[S_\mathrm{sp}]$ remains constant for the entire perfusion period, differentiating equation S12 gives:

$$\frac{dD}{dt} = -K_\mathrm{p}\frac{d[S]}{dt} + K_\mathrm{i}\big([S_\mathrm{sp}] - [S(t)]\big) - K_\mathrm{d}\frac{d^2[S]}{dt^2} \quad \text{for } t_\mathrm{sed} < t. \tag{S13}$$

The second derivative term in equation S13 can be evaluated by differentiating equation S10 with-respect-to t to give:

$$\frac{d^2[S]}{dt^2} = \left([S_\mathrm{I}] - [S(t)]\right)\frac{dD}{dt} - D(t)\frac{d[S]}{dt} - \frac{1}{Y_\mathrm{xs}}\frac{d^2[X]}{dt^2} \quad \text{for } t_\mathrm{sed} < t. \tag{S14}$$

Similarly, the second derivative term on the right-hand-side of equation S14 can be evaluated by differentiating equation S2 with-respect-to t to give:

$$\frac{d^2[X]}{dt^2} = \mu(t)\frac{d[X]}{dt} + [X(t)]\frac{d\mu}{dt} \tag{S15}$$

$$= \left(\mu(t)^2 + \frac{d\mu}{dt}\right)[X(t)]. \tag{S16}$$

Substituting equations S14 and S16 into equation S13 and rearranging gives:

$$\frac{dD}{dt} = \frac{-K_\mathrm{p}\frac{d[S]}{dt} + K_\mathrm{i}\left([S_\mathrm{sp}] - [S(t)]\right) + K_\mathrm{d}\left(D(t)\frac{d[S]}{dt} + \left(\mu(t)^2 + \frac{d\mu}{dt}\right)\frac{[X(t)]}{\mathrm{Y_{xs}}}\right)}{1 + K_\mathrm{d}\left([S_\mathrm{I}] - [S(t)]\right)} \quad \text{for } t_\mathrm{sed} < t. \tag{S17}$$

A combination of equations S10, S11 and S17 provide a set of ODEs that can be solved numerically. Note that equations S10 and S17 are coupled ODEs that must be solved simultaneously. Once solutions of $D(t)$ and $[S(t)]$ have been obtained, equation S11 can be used to solve for $[P(t)]$ separately.

If, instead of a PID controller, the perfusion rate is controlled via a proportional-integral (PI) controller (i.e. $K_\mathrm{d} = 0$ or at least K_d is sufficiently small that the PID controller can be approximated by a PI controller) then equation S17 reduces to:

$$\frac{dD}{dt} = -K_\mathrm{p}\frac{d[S]}{dt} + K_\mathrm{i}\left([S_\mathrm{sp}] - [S(t)]\right) \quad \text{for } t_\mathrm{sed} < t. \tag{S18}$$

For simplicity, equation S18 is utilized in the main text and section 2.1 below. However, the same approach could be implemented with equation S17 if required.

2.1 Discrete solution

Equations S10, S11 and S18 could be solved numerically via a number of different software applications. For example, with the open-source programming language Python, the SciPy package includes the function ‘solve.ivp’ (within the ‘integrate’ sub-module) which can be used to numerically solve a system of ODEs. However, there are physical constraints on the system described here which makes such an approach problematic. Specifically, all three states have a lower bound of zero (i.e. the perfusion rate, glucose concentration and lactate concentration cannot have negative values). As such, a discrete approach is taken here whereby the ODEs given by equations S10, S18 and S11 are approximated by the following forward finite difference equations, respectively:

$$\frac{[S(t_\mathrm{k+1})] - [S(t_\mathrm{k})]}{\Delta t} = D(t_\mathrm{k})\left([S_\mathrm{I}] - [S(t_\mathrm{k})]\right) - \frac{\mu(t_\mathrm{k})[X(t_\mathrm{k})]}{Y_\mathrm{xs}}, \tag{S19}$$

$$\frac{D(t_\mathrm{k+1}) - D(t_\mathrm{k})}{\Delta t} = -K_\mathrm{p}\left(\frac{[S(t_\mathrm{k+1})] - [S(t_\mathrm{k})]}{\Delta t}\right) + K_\mathrm{i}\left([S_\mathrm{sp}] - [S(t_\mathrm{k})]\right), \tag{S20}$$

$$\frac{[P(t_\mathrm{k+1})] - [P(t_\mathrm{k})]}{\Delta t} = \mathrm{Y_{px}}\mu(t_\mathrm{k})[X(t_\mathrm{k})] - D(t_\mathrm{k})[P(t_\mathrm{k})], \tag{S21}$$

where Δt is the time-step and where the initial index is given by $\mathrm{k} = 0$ with $t_0 = t_\mathrm{sed}$. The forward Euler method can now be implemented by rearranging equations S19, S20 and S21 as follows:

$$[S(t_{k+1})] = \max\left(0, [S(t_k)] + \Delta t\left(D(t_k)\left([S_I] - [S(t_k)]\right) - \frac{\mu(t_k)[X(t_k)]}{Y_{xs}}\right)\right), \tag{S22}$$

$$D(t_{k+1}) = \max\left(D_{min}, D(t_k) - K_p\left([S(t_{k+1})] - [S(t_k)]\right) + \Delta t K_i\left([S_{sp}] - [S(t_k)]\right)\right), \tag{S23}$$

$$[P(t_{k+1})] = \max\left(0, [P(t_k)] + \Delta t\left(Y_{px}\mu(t_k)[X(t_k)] - D(t_k)[P(t_k)]\right)\right), \tag{S24}$$

where $D_{min} \geq 0$ is the minimum perfusion rate. Note that the 'max' function allows the physical constraints of the system to be implemented at each time-step. The analytical solutions of $\mu(t)$ and $[X(t)]$ described in section 3 can be used to give solutions for all t_k directly without the need to implement a numerical approach. Such solutions can be used in equations S22 and S24 as required. In a similar manner to the coupled ODEs, equations S22 and S23 must be used incrementally, in that $[S(t_1)]$ obtained via equation S22 is required to obtain $D(t_1)$ via equation S23, which itself is required to obtain $[S(t_2)]$ via equation S22 etc. Note that, as $[S(t_{k+1})]$ and $D(t_{k+1})$ are independent of $[P(t_k)]$, equation S24 can be evaluated separately, providing the required $D(t_k)$ has already been obtained. Alternatively, equation S24 could be removed entirely if a digital shadow of only the glucose concentration and perfusion rate was required. This is because, here, the PI(D) controller acts to maintain the glucose concentration at a set-point and has no direct dependency on the lactate concentration.

3 CELL GROWTH MODELS

In the following sub-sections the generalized logistic model of cell growth first introduced by Richards [1] is considered in the context of the digital shadow.

3.1 Exact solution where initial cell concentration is known

The Richards growth model [1] is given by the ODE in equation S2 where $\mu(t)$ is the specific growth rate which can be given by:

$$\mu(t) = \frac{\beta}{\nu}\left(1 - \left(\frac{[X(t)]}{[K]}\right)^{\nu}\right) \quad \text{for } t \geq t_I \text{ and } \nu \neq 0. \tag{S25}$$

In equation S25, $[K] > 0$ is the carrying capacity (i.e. the maximum possible cell concentration), $\nu \neq 0$ is a dimensionless parameter that affects how the specific growth rate decreases over time and β can be given by:

$$\beta = \mu_{max}\nu\left(\frac{1 + W}{W}\right) \quad \text{for } \nu \neq 0, \tag{S26}$$

where:

$$W = \left(\frac{[K]}{[X_I]}\right)^{\nu} - 1 \quad \text{for } \nu \neq 0, \tag{S27}$$

and where μ_{max} is the initial (and maximum) specific growth rate at time t_I. Using equation S25, equation S2 can be solved to give:

$$[X(t)] = \frac{[K]}{\left(1 + W \exp\left(-\beta\left(t - t_{\mathrm{I}}\right)\right)\right)^{1/\nu}} \quad \text{for } t \geq t_{\mathrm{I}} \text{ and } \nu \neq 0. \tag{S28}$$

Substituting equation S28 into equations S25 and S2 gives the time dependent solution of the specific growth rate and the rate of change of cell concentration, respectively, as:

$$\mu(t) = \frac{\mu_{\max}\left(1 + W\right)\exp\left(-\beta\left(t - t_{\mathrm{I}}\right)\right)}{1 + W \exp\left(-\beta\left(t - t_{\mathrm{I}}\right)\right)} \quad \text{for } t \geq t_{\mathrm{I}} \text{ and } \nu \neq 0, \tag{S29}$$

$$\frac{d[X]}{dt} = \frac{\mu_{\max}[K]\left(1 + W\right)\exp\left(-\beta\left(t - t_{\mathrm{I}}\right)\right)}{\left(1 + W \exp\left(-\beta\left(t - t_{\mathrm{I}}\right)\right)\right)^{(1+\nu)/\nu}} \quad \text{for } t \geq t_{\mathrm{I}} \text{ and } \nu \neq 0. \tag{S30}$$

If the initial cell concentration, $[X_{\mathrm{I}}]$ is experimentally observed at time t_{I} then this leaves three, potentially unknown, parameters in the solution to the Richards model given by equations S28, S29 and S30, namely; $[K]$, ν and $\mu_{\max}$. Some special cases of the Richards model include the logistic or Verhulst model ($\nu = 1$), the Gompertz model ($\nu \to 0$) and the negative exponential model ($\nu = -1$) [3].

3.2 Exact solution where initial and second cell concentrations are known

Let us further assume that at some later time $t_{\mathrm{F}} > t_{\mathrm{I}}$, the cell concentration is experimentally observed as $[X_{\mathrm{F}}]$. This second observation allows us to reduce by one the number of unknown parameters by firstly writing equation S28 as:

$$[X_{\mathrm{F}}] = \frac{[K]}{\left(1 + W \exp\left(-\beta\left(t_{\mathrm{F}} - t_{\mathrm{I}}\right)\right)\right)^{1/\nu}} \quad \text{for } \nu \neq 0. \tag{S31}$$

Re-arranging equation S31 allows β to be written as a function of $[X_{\mathrm{F}}]$ and t_{F}, denoted by β_2, as follows:

$$\beta_2 = -\log\left(\left(\left(\frac{[K]}{[X_{\mathrm{F}}]}\right)^{\nu} - 1\right) \Big/ W\right) \Big/ \left(t_{\mathrm{F}} - t_{\mathrm{I}}\right) \quad \text{for } \nu \neq 0. \tag{S32}$$

Substituting equation S32 into equation S26 allows $\mu_{\max}$ to be written as a function of $[X_{\mathrm{F}}]$ and t_{F}, denoted by $\mu_{\max 2}$, as follows:

$$\mu_{\max 2} = \frac{\beta_2}{\nu}\left(\frac{W}{1 + W}\right) \quad \text{for } \nu \neq 0. \tag{S33}$$

Equations S32 and S33 can now be substituted into equations S28, S29 and S30 to give solutions that are functions of $[X_{\mathrm{F}}]$ and t_{F} (i.e. no longer functions of $\mu_{\max}$), denoted by $[X_2(t)]$, $\mu_2(t)$ and $d[X_2]/dt$, as follows:

$$[X_2(t)] = \frac{[K]}{\left(1 + W\exp\left(-\beta_2\left(t - t_{\mathrm{I}}\right)\right)\right)^{1/\nu}} \quad \text{for } t \geq t_{\mathrm{I}} \text{ and } \nu \neq 0, \tag{S34}$$

$$\mu_2(t) = \frac{\beta_2 W\exp\left(-\beta_2\left(t - t_{\mathrm{I}}\right)\right)}{\nu\left(1 + W\exp\left(-\beta_2\left(t - t_{\mathrm{I}}\right)\right)\right)} \quad \text{for } t \geq t_{\mathrm{I}} \text{ and } \nu \neq 0, \tag{S35}$$

$$\frac{d[X_2]}{dt} = \frac{\beta_2 W[K]\exp\left(-\beta_2\left(t - t_{\mathrm{I}}\right)\right)}{\nu\left(1 + W\exp\left(-\beta_2\left(t - t_{\mathrm{I}}\right)\right)\right)^{(1+\nu)/\nu}} \quad \text{for } t \geq t_{\mathrm{I}} \text{ and } \nu \neq 0. \tag{S36}$$

In summary, if t_{I}, $[X_{\mathrm{I}}]$, t_{F} and $[X_{\mathrm{F}}]$ are known then there remain two, potentially unknown, parameters in the solution to the Richards model given by equations S34, S35 and S36, namely $[K]$ and ν.

3.3 Exact solution with a lag period where initial cell concentration is known

In this section the solutions described in section 3.1 are extended to include an initial lag period of duration $t_{\mathrm{lag}} - t_{\mathrm{I}} > 0$ in which there is no cell growth. The effect of the lag period is to time-delay equations S28, S29 and S30 by the same period as follows:

$$[X_{\mathrm{lag}}(t)] = \frac{[K]}{\left(1 + W\exp\left(-H\left(t - t_{\mathrm{lag}}\right)\beta\left(t - t_{\mathrm{lag}}\right)\right)\right)^{1/\nu}} \quad \text{for } t \geq t_{\mathrm{I}} \text{ and } \nu \neq 0, \tag{S37}$$

$$\mu_{\mathrm{lag}}(t) = \frac{H\left(t - t_{\mathrm{lag}}\right)\mu_{\mathrm{max}}\left(1 + W\right)\exp\left(-\beta\left(t - t_{\mathrm{lag}}\right)\right)}{1 + W\exp\left(-H\left(t - t_{\mathrm{lag}}\right)\beta\left(t - t_{\mathrm{lag}}\right)\right)} \quad \text{for } t \geq t_{\mathrm{I}} \text{ and } \nu \neq 0, \tag{S38}$$

$$\frac{d[X_{\mathrm{lag}}]}{dt} = \frac{H\left(t - t_{\mathrm{lag}}\right)\mu_{\mathrm{max}}[K]\left(1 + W\right)\exp\left(-\beta\left(t - t_{\mathrm{lag}}\right)\right)}{\left(1 + W\exp\left(-H\left(t - t_{\mathrm{lag}}\right)\beta\left(t - t_{\mathrm{lag}}\right)\right)\right)^{(1+\nu)/\nu}} \quad \text{for } t \geq t_{\mathrm{I}} \text{ and } \nu \neq 0, \tag{S39}$$

If $[X_{\mathrm{I}}]$ and t_{I} are known then this leaves four, potentially unknown, parameters in equations S37, S38 and S39, namely; $[K]$, ν, μ_{max} and t_{lag}.

3.4 Exact solution with a lag period where initial and second cell concentrations are known

In this section, the solutions described in section 3.2 are extended to include the same lag period that was introduced in sub-section 3.3. The effect of the lag period is to time-delay equations S34, S35 and S36 as follows:

$$[X_{2\mathrm{lag}}(t)] = \frac{[K]}{\left(1 + W\exp\left(-H\left(t - t_{\mathrm{lag}}\right)\beta_{2\mathrm{lag}}\left(t - t_{\mathrm{lag}}\right)\right)\right)^{1/\nu}} \quad \text{for } t \geq t_{\mathrm{I}} \text{ and } \nu \neq 0, \tag{S40}$$

$$\mu_{2\mathrm{lag}}(t) = \frac{H\left(t - t_{\mathrm{lag}}\right)\beta_{2\mathrm{lag}}W\exp\left(-\beta_{2\mathrm{lag}}\left(t - t_{\mathrm{lag}}\right)\right)}{\nu\left(1 + W\exp\left(-H\left(t - t_{\mathrm{lag}}\right)\beta_{2\mathrm{lag}}\left(t - t_{\mathrm{lag}}\right)\right)\right)} \quad \text{for } t \geq t_{\mathrm{I}} \text{ and } \nu \neq 0, \tag{S41}$$

$$\frac{d[X_{2\mathrm{lag}}]}{dt} = \frac{H\left(t - t_{\mathrm{lag}}\right)\beta_{2\mathrm{lag}}W[K]\exp\left(-\beta_{2\mathrm{lag}}\left(t - t_{\mathrm{lag}}\right)\right)}{\nu\left(1 + W\exp\left(-H\left(t - t_{\mathrm{lag}}\right)\beta_{2\mathrm{lag}}\left(t - t_{\mathrm{lag}}\right)\right)\right)^{(1+\nu)/\nu}} \quad \text{for } t \geq t_{\mathrm{I}} \text{ and } \nu \neq 0, \tag{S42}$$

where:

$$\beta_{2\text{lag}} = -\log\left(\left(\left(\frac{[K]}{[X_{2\text{lagF}}]}\right)^{\nu} - 1\right) \Big/ W\right) \Big/ (t_{\text{F}} - t_{\text{lag}}) \quad \text{for } t_{\text{F}} > t_{\text{lag}} \text{ and } \nu \neq 0. \tag{S43}$$

If t_{I}, $[X_{\text{I}}]$, t_{F} and $[X_{2\text{lagF}}]$ are known then there remain three, potentially unknown, parameters in equations S40, S41 and S42, namely: $[K]$, ν, and t_{lag}. Note that it is assumed that $t_{\text{F}} > t_{\text{lag}}$ and, therefore, $[X_{2\text{lagF}}] > [X_{\text{I}}]$.

3.5 Approximate solution where initial cell concentration is known

The specific growth rate given by equation S25 can be re-written in the following form:

$$\mu(t) = \mu_{\max}\left(\frac{[K]^{\nu} - [X(t)]^{\nu}}{[K]^{\nu} - [X_{\text{I}}]^{\nu}}\right) \quad \text{for } t \geq t_{\text{I}} \text{ and } \nu \neq 0. \tag{S44}$$

If $[K]^{\nu} \ll [X(t)]^{\nu}$ and $[K]^{\nu} \ll [X_{\text{I}}]^{\nu}$ then equation S44 can be approximated by:

$$\mu(t) \approx \mu_{\max}\left(\frac{[X(t)]}{[X_{\text{I}}]}\right)^{\nu} = \mu_{\text{app}}(t) \quad \text{for } t \geq t_{\text{I}} \text{ and } \nu \neq 0. \tag{S45}$$

Given that $[X_{\text{I}}] \leq [X(t)] < [K]$ for $t_{\text{I}} \leq t$, the two requirements for equation S45 to be valid are $\nu < 0$ and $[X(t)]^{-\nu} \ll [K]^{-\nu}$. For example, with the negative exponential model, equation S45 reduces to:

$$\mu_{\text{app}}(t) = \frac{\mu_{\max}[X_{\text{I}}]}{[X(t)]} \quad \text{for } \nu = -1 \text{ and } [X(t)] \ll [K]. \tag{S46}$$

Substituting equation S46 into equation S2 gives:

$$\frac{d[X_{\text{app}}]}{dt} = \mu_{\max}[X_{\text{I}}] \quad \text{for } t \geq t_{\text{I}} \tag{S47}$$

$$\Rightarrow [X_{\text{app}}(t)] = [X_{\text{I}}]\left(1 + \mu_{\max}(t - t_{\text{I}})\right) \quad \text{for } t \geq t_{\text{I}} \tag{S48}$$

$$\Rightarrow \mu_{\text{app}}(t) = \frac{\mu_{\max}}{1 + \mu_{\max}(t - t_{\text{I}})} \quad \text{for } t \geq t_{\text{I}}, \tag{S49}$$

where $[X_{\text{app}}(t)]$ is the approximate cell concentration. Equations S47, S48 and S49 show that in the early stages of the negative exponential model, the approximate cell concentration increases linearly at a constant rate given by $\mu_{\max}[X_{\text{I}}]$. Another noteworthy example is when $\nu = -1/2$ and $\sqrt{[X(t)]} \ll \sqrt{[K]}$. In this scenario, the cell concentration is given by:

$$[X_{\text{app}}(t)] = [X_{\text{I}}]\left(1 + \frac{\mu_{\max}}{2}(t - t_{\text{I}})\right)^{2} \quad \text{for } t \geq t_{\text{I}}. \tag{S50}$$

Equation S50 shows that in these conditions the cells undergo approximately quadratic growth. More generally though, for $\nu < 0$ and $[X(t)]^{-\nu} \ll [K]^{-\nu}$, substituting equation S45 into equation S2 gives:

$$\frac{d[X_{\text{app}}]}{dt} = \frac{\mu_{\text{max}}}{[X_{\text{I}}]^{\nu}}[X_{\text{app}}(t)]^{1+\nu}. \tag{S51}$$

Equation S51 can be solved to give:

$$[X_{\text{app}}(t)] = \frac{[X_{\text{I}}]}{(1-\mu_{\text{max}}\nu\,(t-t_{\text{I}}))^{1/\nu}} \quad \text{for } t \geq t_{\text{I}} \text{ and } \nu < 0, \tag{S52}$$

$$\mu_{\text{app}}(t) = \frac{\mu_{\text{max}}}{1-\mu_{\text{max}}\nu\,(t-t_{\text{I}})} \quad \text{for } t \geq t_{\text{I}} \text{ and } \nu < 0, \tag{S53}$$

$$\frac{d[X_{\text{app}}]}{dt} = \frac{\mu_{\text{max}}[X_{\text{I}}]}{(1-\mu_{\text{max}}\nu\,(t-t_{\text{I}}))^{\frac{1+\nu}{\nu}}} \quad \text{for } t \geq t_{\text{I}} \text{ and } \nu < 0. \tag{S54}$$

If t_{I} and $[X_{\text{I}}]$ are known and if it can be assumed that $\nu < 0$ and $[X(t)]^{-\nu} \ll [K]^{-\nu}$, then this leaves two, potentially unknown, parameters in the approximate solution given by equations S52, S53 and S54, namely; μ_{max} and ν. Therefore, under these conditions the approximate solutions are independent of the carrying capacity, $[K]$ (*cf* section 3.1).

It is worth emphasizing that although there is a requirement for $\nu < 0$, as $\nu \to 0$ equations S52, S53 and S54 tend to those of exponential growth. This can be realized by simply substituting $\nu = 0$ into equation S51 to give the standard ODE of exponential growth. Clearly, such a substitution invalidates the requirement for $[X(t)]^{-\nu} \ll [K]^{-\nu}$ under which such approximated equations hold. However, in the early stages of cell growth and in the absence of knowledge of (or the ability to fit - see section 4 below) the carrying capacity, $[K]$, equations S52, S53 and S54 capture a range of cell growth patterns, including (but not limited to) linear, quadratic and exponential growth. These equations are also appealing because the early stages of logistic growth ($\nu = 1$) are approximately exponential. Thus, equations S52, S53 and S54 offer both simple and flexible two parameter functions of cell growth that are also based on mechanistic considerations.

3.6 Approximate solution where initial and second cell concentrations are known

In a similar manner to section 3.2, let us further assume that a cell concentration $[X_{\text{appF}}]$ is known at some later time $t_{\text{F}} > t_{\text{I}}$. This additional knowledge allows us to reduce by one the number of unknown parameters by firstly writing equation S52 as:

$$[X_{\text{appF}}] = \frac{[X_{\text{I}}]}{(1-\mu_{\text{max}}\nu\,(t_{\text{F}}-t_{\text{I}}))^{1/\nu}} \quad \text{for } \nu < 0. \tag{S55}$$

Rearranging equation S55 μ_{max} to be written as a function of $[X_{\text{appF}}]$ and t_{F}, denoted by μ_{max2app}, as follows:

$$\mu_{\text{max2app}} = \left(1-\left(\frac{[X_{\text{I}}]}{[X_{\text{appF}}]}\right)^{\nu}\right) \Big/ \left(\nu\,(t_{\text{F}}-t_{\text{I}})\right) \quad \text{for } \nu < 0. \tag{S56}$$

Equation S56 can now be substituted into equations S52, S53 and S54 to give solutions that are functions of $[X_{\text{appF}}]$ and t_{F} (i.e. no longer functions of μ_{max}):

$$[X_{\text{app2}}(t)] = \frac{[X_{\text{I}}]}{\left(1 - \mu_{\text{max2app}}\nu\,(t - t_{\text{I}})\right)^{1/\nu}} \quad \text{for } t \geq t_{\text{I}} \text{ and } \nu < 0, \tag{S57}$$

$$\mu_{\text{app2}}(t) = \frac{\mu_{\text{max2app}}}{1 - \mu_{\text{max2app}}\nu\,(t - t_{\text{I}})} \quad \text{for } t \geq t_{\text{I}} \text{ and } \nu < 0, \tag{S58}$$

$$\frac{d[X_{\text{app2}}]}{dt} = \frac{\mu_{\text{max2app}}[X_{\text{I}}]}{\left(1 - \mu_{\text{max2app}}\nu\,(t - t_{\text{I}})\right)^{\frac{1+\nu}{\nu}}} \quad \text{for } t \geq t_{\text{I}} \text{ and } < 0. \tag{S59}$$

If t_{I}, $[X_{\text{I}}]$, t_{F} and $[X_{\text{appF}}]$ are known then there remains one, potentially unknown, parameter in equations S57, S58 and S59, namely: ν.

3.7 Approximate solution with a lag period where initial cell concentration is known

In a similar manner to section 3.3, the solutions described in section 3.5 are extended to include an initial lag period of duration $t_{\text{lag}} - t_{\text{I}} > 0$ in which there is no cell growth. The effect of the lag period is to time-delay equations S52, S53 and S54 by the same period as follows:

$$[X_{\text{applag}}(t)] = \frac{[X_{\text{I}}]}{\left(1 - H\left(t - t_{\text{lag}}\right)\mu_{\text{max}}\nu\,(t - t_{\text{lag}})\right)^{1/\nu}} \quad \text{for } t \geq t_{\text{I}} \text{ and } \nu < 0, \tag{S60}$$

$$\mu_{\text{applag}}(t) = \frac{H\left(t - t_{\text{lag}}\right)\mu_{\text{max}}}{1 - H\left(t - t_{\text{lag}}\right)\mu_{\text{max}}\nu\,(t - t_{\text{lag}})} \quad \text{for } t \geq t_{\text{I}} \text{ and } \nu < 0, \tag{S61}$$

$$\frac{d[X_{\text{applag}}]}{dt} = \frac{H\left(t - t_{\text{lag}}\right)\mu_{\text{max}}[X_{\text{I}}]}{\left(1 - H\left(t - t_{\text{lag}}\right)\mu_{\text{max}}\nu\,(t - t_{\text{lag}})\right)^{\frac{1+\nu}{\nu}}} \quad \text{for } t \geq t_{\text{I}} \text{ and } \nu < 0. \tag{S62}$$

If the initial cell concentration, $[X_{\text{I}}]$ is experimentally observed at time t_{I} then this leaves three, potentially unknown, parameters in equations S60, S61 and S62, namely; ν, μ_{max} and t_{lag}.

3.8 Approximate solution with a lag period where initial and second cell concentrations are known

In this section, the solutions described in section 3.6 are extended to include the same lag period that was introduced in sub-section 3.7. The effect of the lag period is to time-delay equations S57, S58 and S59 as follows:

$$[X_{\text{app2lag}}(t)] = \frac{[X_{\text{I}}]}{\left(1 - H\left(t - t_{\text{lag}}\right)\mu_{\text{max2applag}}\nu\left(t - t_{\text{lag}}\right)\right)^{1/\nu}} \quad \text{for } t \geq t_{\text{I}} \text{ and } \nu < 0, \tag{S63}$$

$$\mu_{\text{app2lag}}(t) = \frac{H\left(t - t_{\text{lag}}\right)\mu_{\text{max2applag}}}{1 - H\left(t - t_{\text{lag}}\right)\mu_{\text{max2applag}}\nu\left(t - t_{\text{lag}}\right)} \quad \text{for } t \geq t_{\text{I}} \text{ and } \nu < 0, \tag{S64}$$

$$\frac{d[X_{\text{app2lag}}]}{dt} = \frac{H\left(t - t_{\text{lag}}\right)\mu_{\text{max2applag}}[X_{\text{I}}]}{\left(1 - H\left(t - t_{\text{lag}}\right)\mu_{\text{max2applag}}\nu\left(t - t_{\text{lag}}\right)\right)^{\frac{1+\nu}{\nu}}} \quad \text{for } t \geq t_{\text{I}} \text{ and } \nu < 0, \tag{S65}$$

where:

$$\mu_{\text{max2applag}} = \left(1 - \left(\frac{[X_{\text{I}}]}{[X_{\text{appFlag}}]}\right)^{\nu}\right) \Big/ \left(\nu\left(t_{\text{F}} - t_{\text{lag}}\right)\right) \quad \text{for } t_{\text{F}} > t_{\text{lag}} \text{ and } \nu < 0. \tag{S66}$$

If t_{I}, $[X_{\text{I}}]$, t_{F} and $[X_{\text{appFlag}}]$ are known then there remain two, potentially unknown, parameters in equations S63, S64 and S65, namely: ν, and t_{lag}. Note that it is assumed that $t_{\text{F}} > t_{\text{lag}}$ and, therefore, $[X_{\text{appFlag}}] > [X_{\text{I}}]$.

3.9 Approximate confidence bands

In this section confidence bands for the approximate cell concentration and approximate specific growth rate are derived based on the method described here [4]. This method is also recommended (and partially implemented) by the lmfit package that was used to fit the digital shadow to the data [5]. The derivation of the confidence bands that follows is based on the assumption that t_{lag} is a fixed parameter (i.e. that it is not a fitted parameter - see section 4.2 for details). Utilizing equation S60 it can be shown that the partial derivatives of the approximate cell concentration are given by:

$$\frac{\partial[X_{\text{applag}}]}{\partial\mu_{\text{max}}} = [X_{\text{applag}}(t)]\left(\frac{H(t - t_{\text{lag}})(t - t_{\text{lag}})}{1 - H(t - t_{\text{lag}})\mu_{\text{max}}\nu(t - t_{\text{lag}})}\right) \tag{S67}$$

$$\frac{\partial[X_{\text{applag}}]}{\partial\nu} = \frac{[X_{\text{applag}}(t)]}{\nu^2}\left(\log\left(1 - H(t - t_{\text{lag}})\mu_{\text{max}}\nu(t - t_{\text{lag}})\right) + \frac{H(t - t_{\text{lag}})\mu_{\text{max}}\nu(t - t_{\text{lag}})}{1 - H(t - t_{\text{lag}})\mu_{\text{max}}\nu(t - t_{\text{lag}})}\right) \tag{S68}$$

Similarly, utilizing equation S61 it can be shown that the partial derivatives of the approximate specific growth rate are given by:

$$\frac{\partial\mu_{\text{applag}}}{\partial\mu_{\text{max}}} = \left(\frac{\mu_{\text{applag}}(t)}{\mu_{\text{max}}}\right)^2 \tag{S69}$$

$$\frac{\partial\mu_{\text{applag}}}{\partial\nu} = \left(\mu_{\text{applag}}(t)\right)^2\left(t - t_{\text{lag}}\right) \tag{S70}$$

Equations S67, S68, S69 and S70 are required in the following equation:

$$\Delta_f = \left(\frac{\partial f}{\partial \mu_{\text{max}}}\right)^2 \text{cov}[\mu_{\text{max}}, \mu_{\text{max}}] + \frac{\partial f}{\partial \mu_{\text{max}}}\frac{\partial f}{\partial \nu}\text{cov}[\mu_{\text{max}}, \nu] + \frac{\partial f}{\partial \nu}\frac{\partial \mu}{\partial \mu_{\text{max}}}\text{cov}[\nu, \mu_{\text{max}}] + \left(\frac{\partial f}{\partial \nu}\right)^2 \text{cov}[\nu, \nu] \tag{S71}$$

where $f = \{[X_{\text{applag}}(t)], \mu_{\text{applag}}(t)\}$ and $\text{cov}[i, j]$ is the i, j entry of covariance matrix resulting from the fitting process. Using equation S71, the confidence bounds, B are given by:

$$B = \hat{f} \pm \tau_{\alpha/2,n}\sqrt{\chi_n^2 \Delta_f} \tag{S72}$$

where $\hat{f}$ is the value of f with the best-fitted parameters, n is the degrees of freedom (i.e. the number of data points used to fit the model minus the number of parameters to be fitted), χ_n^2 is the reduced chi-squared statistic resulting from the fitting process and $\tau_{\alpha/2,n}$ is the upper $\alpha/2$ critical value of Student's t distribution. In the main text $\alpha = 0.05$ which results in a 95% confidence band around the best-fit predictions.

4 IDENTIFIABILITY ANALYSIS

Model parameters that are structurally unidentifiable can be replaced by an infinite number of values without effecting the model output. Therefore, any parameter estimation obtained for a structurally unidentifiable parameter would be deemed unreliable. In this section structural identifiability analysis is performed to establish if meaningful parameter estimates can be obtained for the digital shadow described in sections 1, 2 and 3. Although the analysis that follows is applicable to equations S3 (glucose concentration), S4 (lactate concentration) and S18 (perfusion rate), the results also hold with the addition of the normalized DO equation described in the main text.

4.1 Application to digital shadow

There are many different methods that can be used to assess the structural identifiability of a model such as the Taylor series method, the Laplace method and the input-output method [6]. Here the exact arithmetic rank (EAR) method [7, 8] is used to assess the structural identifiability of the digital shadow. The EAR method is available as a package in Mathematica [9] which is freely available for research purposes upon request. In the following analysis, the digital shadow based on the cell growth model in section 3.6 (equations S56 and S59), is assessed for structural identifiability via the EAR Mathematica tool. However, before doing so, a number of modifications must be made to the digital shadow. First, it is assumed that $t_{\text{sed}} < t$ and, therefore, $H(t - t_{\text{sed}}) = 1$. Second, it is necessary to make the following reparameterization:

$$[X_{\text{IF}\nu}] = \left(\frac{[X_{\text{I}}]}{[X_{\text{appF}}]}\right)^{\nu}. \tag{S73}$$

The right hand side of equation S73 needs to be treated as a single parameter in the EAR tool due to the unknown parameter ν in the exponent. Substituting equation S73 into equation S59 gives:

$$\frac{d[X_{\text{app2}}]}{dt} = \frac{[X_{\text{I}}](1 - [X_{\text{IF}\nu}])\left(1 - \frac{(t-t_{\text{I}})(1-[X_{\text{IF}\nu}])}{t_{\text{F}}-t_{\text{I}}}\right)^{-\frac{1+\nu}{\nu}}}{\nu(t_{\text{F}} - t_{\text{I}})}. \tag{S74}$$

Third, two further time-dependent reparameterizations are required as follows, again due to the unknown parameter ν in the exponent:

$$\alpha(t) = 1 - \frac{(t - t_{\mathrm{I}})(1 - [X_{\mathrm{IF}\nu}])}{t_{\mathrm{F}} - t_{\mathrm{I}}}, \tag{S75}$$

$$\gamma(t) = \alpha(t)^{-\frac{1+\nu}{\nu}}. \tag{S76}$$

Fourth, for the analysis in this section the initial conditions of equations S75 and S76 are given by:

$$\alpha(t_{\mathrm{I}}) = 1, \tag{S77}$$

$$\gamma(t_{\mathrm{I}}) = 1. \tag{S78}$$

Fifth, equations S75 and S76 are state variables which means their derivatives are also required:

$$\frac{d\alpha}{dt} = -\left(\frac{1 - [X_{\mathrm{IF}\nu}]}{t_{\mathrm{F}} - t_{\mathrm{I}}}\right), \tag{S79}$$

$$\frac{d\gamma}{dt} = \frac{(1+\nu)(1 - [X_{\mathrm{IF}\nu}])\gamma(t)}{\nu(t_{\mathrm{F}} - t_{\mathrm{I}})\alpha(t)}. \tag{S80}$$

The observed states are $D(t)$, $[S(t)]$ and $[P(t)]$ and the known parameters are t_{I}, t_{F}, $[X_{\mathrm{I}}]$, K_{p}, K_{i}, $[S_{\mathrm{I}}]$, $[S_{\mathrm{sp}}]$. Note that $[X_{\mathrm{appF}}]$ is also a known parameter although this parameter is only present implicitly in $[X_{\mathrm{IF}\nu}]$ via equation S73. Therefore, there are three unknown parameters which are Y_{xs}, Y_{px} and ν. The black Mathematica code below shows the input commands used in the EAR tool. The red text that follows shows the corresponding output from the EAR tool.

```
Needs["IdentifiabilityAnalysis`"]
modelStates = {X,Dil,S,P,a,g};
deq = {
 X'[t]==XI*(1-XIFv)*g[t]/(v*(tF-tI)),
 Dil'[t] == Ki*(Ssp-S[t]) -
 Kp*(Dil[t]*(SI-S[t]) - (XI*(1-XIFv)*g[t]/(Yxs*v*(tF-tI)))),
 S'[t] == Dil[t]*(SI-S[t]) - (XI*(1-XIFv)*g[t]/(Yxs*v*(tF-I))),
 P'[t] == -Dil[t]*P[t] + (Ypx*XI*(1-XIFv)*g[t]/(v*(tF-tI))),
 a'[t] == -(1-XIFv)/(tF-I),
 g'[t] == (1+v)*(1-XIFv)*g[t]/(v*(tF-tI)*a[t])
};
ic={
```

```
 X[0]==XI,
 Dil[0]==0,
 S[0]==25,
 P[0]==0,
 a[0]==1,
 g[0]==1
};
modelParameters = {tI,tF,XI,Kp,Ki,SI,Ssp,Yxs,Ypx,v,XIFv};
observationVector={Dil[t],S[t],P[t],tI,tF,XI,Kp,Ki,SI,Ssp};
iad=IdentifiabilityAnalysis[
 {{deq,ic},observationVector},modelStates,modelParameters,t
];
iad["IdentifiableQ"]
```

```
True
```

The output from the EAR tool above indicates that the digital shadow that includes the cell growth model described in section 3.6 is structurally identifiable.

4.2 Application to bioreactor run data

Table S1 provides a summary of output from the EAR tool for the digital shadow as stratified by the cell growth models described in sections 3.1 - 3.8. Of the four models that utilize knowledge of two cell concentrations at two different times, three are structurally identifiable (models 3.2, 3.6 and 3.8) and one is structurally unidentifiable (model 3.4). Table S2 shows goodness-of-fit measures, as assessed by Akaike's Information Criterion (AIC), for the three structurally identifiable models when fitted to the data from the five Aglaris FACER runs (i.e. the five training datasets). It can be seen via the similarity in the AIC values that for runs 1 and 2, the approximate model 3.6 (which does not include the carrying capacity as a parameter) provides a similar goodness-of-fit to the exact model 3.2. Furthermore, for runs 2 and 5, the best-fitted carrying capacities with the exact model 3.2 are extremely large (arguably physically unrealistic) and the associated confidence intervals are also very large. Although not a formal assessment of practical identifiability, the highly uncertain estimates of the carrying capacity suggest that fitting model 3.2 to such bioreactor data is problematic and should be used with caution. For run 4 where the exact model 3.2 gives an improved fit over the approximate model 3.6, the fitted carrying capacity is lower than the measured cell concentration at the end of the expansion period for runs 1, 2, 3 and 6 (see main text). This casts further doubt on the accuracy of the best-fitted carrying capacities because, by definition, the carrying capacity is at least as large as any measured cell concentration for a given bioreactor. In addition, the fitting process is unsuccessful when attempting to fit the exact model 3.2 to the data from run 5. Collectively, these results suggest that the approximate cell growth model 3.6 is more appropriate to use in the digital shadow than the exact cell growth model 3.2.

Table S2 also shows that the approximate model 3.8 (which includes a lag period) gives improved fits for runs 1, 2 and 4 compared with the approximate model 3.6 (where there is no lag period). However, only

with run 1 does the inclusion of a lag period lead to a considerable improvement in model fit. With runs 2 and 4, the improvement in model fit is marginal. In runs 5 and 6 where there is no improvement in model fit by including a lag period (i.e. the AIC values are effectively the same) the predicted lag period is zero days. Collectively, these results suggest that the approximate model 3.8 (which includes a lag period) can better explain the data for some bioreactor runs but often only provides a marginal improvement. Given that table S1 shows that model 3.7 (i.e. the real-time version of model 3.8) is structurally identifiable only if there is prior knowledge of either the maximum specific growth rate or the lag period, the analysis here provides justification for fixing the lag period (at the average of the fitted values from the digital shadow training) for the digital shadow testing in the main text.

5 SUPPLEMENTARY TABLES

Cell growth model	Number of unknown parameters	Structurally identifiable?	Parameters required for identifiability
3.1	5	Yes	
3.2	4	Yes	
3.3	6	No	μ_{max} or t_{lag} or $[K]$
3.4	5	No	t_{lag} or $[K]$
3.5	4	Yes	
3.6	3	Yes	
3.7	5	No	μ_{max} or t_{lag}
3.8	4	Yes	

Table S1. Structural identifiability analysis summary of all versions of the digital shadow. Note that the total number of unknown parameters is the number of unknown parameters in the cell growth models 3.1-3.8 plus the two constituent growth yield factors described in section 1. The EAR method can only show that model 3.3 is identifiable with knowledge of either μ_{max} or $[K]$. The Taylor series method was used to show that model 3.3 is identifiable with knowledge of t_{lag}.

Run	Measure	Model 3.2	Model 3.6	Model 3.8
1	AIC	-45,453	-44,793	-61,572
	$[K]$	1.07E+02 (9.82E+01, 1.16E+02)		
	$t_{\text{lag}} - t_{\text{I}}$			0.160 (0.158,0.161)
2	AIC	-70,584	-70,586	-71,338
	$[K]$	1.67E+13 (0,3.84E+16)		
	$t_{\text{lag}} - t_{\text{I}}$			0.172 (0.164, 0.181)
4	AIC	-42,101	-29,758	-30,502
	$[K]$	1.46E+01 (1.45E+01, 1.47E+01)		
	$t_{\text{lag}} - t_{\text{I}}$			0.128 (0.119, 0.137)
5	AIC	Unsuccessful convergence in fitting process	-22,453	-22,451
	$[K]$			
	$t_{\text{lag}} - t_{\text{I}}$			0.00 (0.000, 0.0188)
6	AIC	112,940	120,104	120,106
	$[K]$	4.81E+07 (0, 5.71E+11)		
	$t_{\text{lag}} - t_{\text{I}}$			0.00 (0.00, 0.350)

Table S2. Comparison of the structurally identifiable digital shadow fits for the five Aglaris FACER training runs. 'AIC' is an acronym for 'Akaike's Information Criterion'. $[K]$ is the carrying capacity with units of 10^6 cells/mL. $t_{\text{lag}} - t_{\text{I}}$ is the lag period with units of days. Values in parentheses are the 95% confidence intervals.

	Initial parameter values				Key outputs of fitting process				
Run	Y_{xs}	Y_{px}	t_{lag} - t_I	ν	No. of func. evals.	χ^2	χ^2_n	AIC	Best-fit match?
1	0.1	15	0.05	-0.1	48	73,747	0.60	-61,572	Yes
	0.1	15	0.05	-2.0	36	73,747	0.60	-61,572	Yes
	1.0	15	0.05	-0.1	62	1,711,960	14.0	322,439	No
	0.1	2.0	0.05	-0.1	46	73,747	0.60	-61,572	Yes
	0.1	15	0.3	-0.1	52	73,747	0.60	-61,572	Yes
	1.0	2.0	0.05	-0.1	116	73,747	0.60	-61,572	Yes
	1.0	2.0	0.3	-2.0	125	124,891	1.02	2,756	No
	1.0	2.0	0.05	-2.0	304	138,724	1.14	15,583	No
2	0.1	15	0.05	-0.1	106	75,041	0.58	-71,338	Yes
	0.1	15	0.05	-2.0	78	75,041	0.58	-71,338	Yes
	1.0	15	0.05	-0.1	105	75,041	0.58	-71,338	Yes
	0.1	2.0	0.05	-0.1	119	75,041	0.58	-71,338	Yes
	0.1	15	0.3	-0.1	80	75,041	0.58	-71,338	Yes
	1.0	2.0	0.05	-0.1	231	75,041	0.58	-71,338	Yes
	1.0	2.0	0.3	-2.0	116	75,041	0.58	-71,338	Yes
	1.0	2.0	0.05	-2.0	73	75,041	0.58	-71,338	Yes
4	0.1	15	0.05	-0.1	56	81,148	0.75	-30,502	Yes
	0.1	15	0.05	-2.0	169	349,438	3.2	126,771	No
	1.0	15	0.05	-0.1	241	1,734,394	16.1	299,345	No
	0.1	2.0	0.05	-0.1	70	81,148	0.75	-30,502	Yes
	0.1	15	0.3	-0.1	46	81,148	0.75	-30,502	Yes
	1.0	2.0	0.05	-0.1	35	339,549	3.15	123,679	No
	1.0	2.0	0.3	-2.0	86	81,148	0.75	-30,502	Yes
	1.0	2.0	0.05	-2.0	108	81,148	0.75	-30,502	Yes
5	0.1	15	0.05	-0.1	96	84,354	0.81	-22,451	Yes
	0.1	15	0.05	-2.0	124	84,354	0.81	-22,451	Yes
	1.0	15	0.05	-0.1	623	84,354	0.81	-22,451	Yes
	0.1	2.0	0.05	-0.1	91	84,354	0.81	-22,451	Yes
	0.1	15	0.3	-0.1	91	84,354	0.81	-22,451	Yes
	1.0	2.0	0.05	-0.1	249	84,354	0.81	-22,451	Yes
	1.0	2.0	0.3	-2.0	173	84,354	0.81	-22,451	Yes
	1.0	2.0	0.05	-2.0	185	84,354	0.81	-22,451	Yes
6	0.1	15	0.05	-0.1	84	329,967	3.17	120,106	Yes
	0.1	15	0.05	-2.0	102	329,967	3.17	120,106	Yes
	1.0	15	0.05	-0.1	2,108	558,356	5.4	174,877	No
	0.1	2.0	0.05	-0.1	89	329,967	3.17	120,106	Yes
	0.1	15	0.3	-0.1	114	329,967	3.17	120,106	Yes
	1.0	2.0	0.05	-0.1	137	329,967	3.17	120,106	Yes
	1.0	2.0	0.3	-2.0	293	329,967	3.17	120,106	Yes
	1.0	2.0	0.05	-2.0	139	329,967	3.17	120,106	Yes

Table S3. Sensitivity analysis of the initial parameter values. The lower and upper bounds of the fitted parameters are given in parentheses as follows: Y_{xs} (0, ∞), Y_{px} (0, ∞), t_{lag} - t_I (0, t_F - t_I) and ν ($-\infty$, 0).

Measure	Run 1	Run 2	Run 4	Run 5	Run 6	Mean	|CV|
Y_{xs}	0.231	0.275	0.272	0.207	0.311	0.259	0.140
	(0.230, 0.231)	(0.275 , 0.276)	(0.271,0.273)	(0.206, 0.208)	(0.309, 0.313)		
Y_{px}	7.93	6.25	9.09	6.83	7.99	7.62	0.130
	(7.91, 7.94)	(6.23, 6.26)	(9.06, 9.13)	(6.80, 6.87)	(7.95, 8.04)		
$t_{lag} - t_I$	0.160	0.167	0.128	0.00	0.00	0.091	0.829
	(0.158, 0.161)	(0.160, 0.175)	(0.120, 0.135)	(0.00, 0.042)	(0.00, 0.006)		
ν	-0.922	-0.475	-0.524	-0.00	-0.761	-0.536	0.584
	(-0.925 , -0.919)	(-0.478, -0.472)	(-0.529, -0.518)	(-0.008, -0.00)	(-0.774, -0.749)		
$k_L a$	3.00	741	1.73	42.9	20.2	162	1.79
	(2.11, 3.88)	(0.00, 8.11×10^3)	(0.703, 2.75)	(0.00, 130)	(0.00, 122)		
Y'_{xc}	3.68	0.0220	6.27	0.189	0.893	2.21	1.09
	(3.16, 4.20)	(0.00, 0.239)	(4.56, 7.98)	(0.00, 0.557)	(0.00, 4.92)		
χ^2_n	0.45	0.43	0.57	0.61	2.38		

Table S4. Best-fit parameters with 95% confidence intervals in parentheses of extended model that additionally to the normalized DO data. The initial parameter values of Y_{xs}, Y_{px}, $t_{lag} - t_I$, and ν are given by the average of the fitted values of the standard model in the main text. The initial values of the additional parameters are given by $k_L a = 1$ day^{-1} and $Y'_{xc} = 1 \times 10^6$ cells/mL. The lower and upper bounds of the fitted parameters are given in parentheses as follows: Y_{xs} (0, ∞), Y_{px} (0, ∞), t_{lag} - t_I (0, t_F - t_I), ν ($-\infty$, 0), $k_L a$ (0, ∞) and Y'_{xc} (0, ∞).

6 SUPPLEMENTARY FIGURES

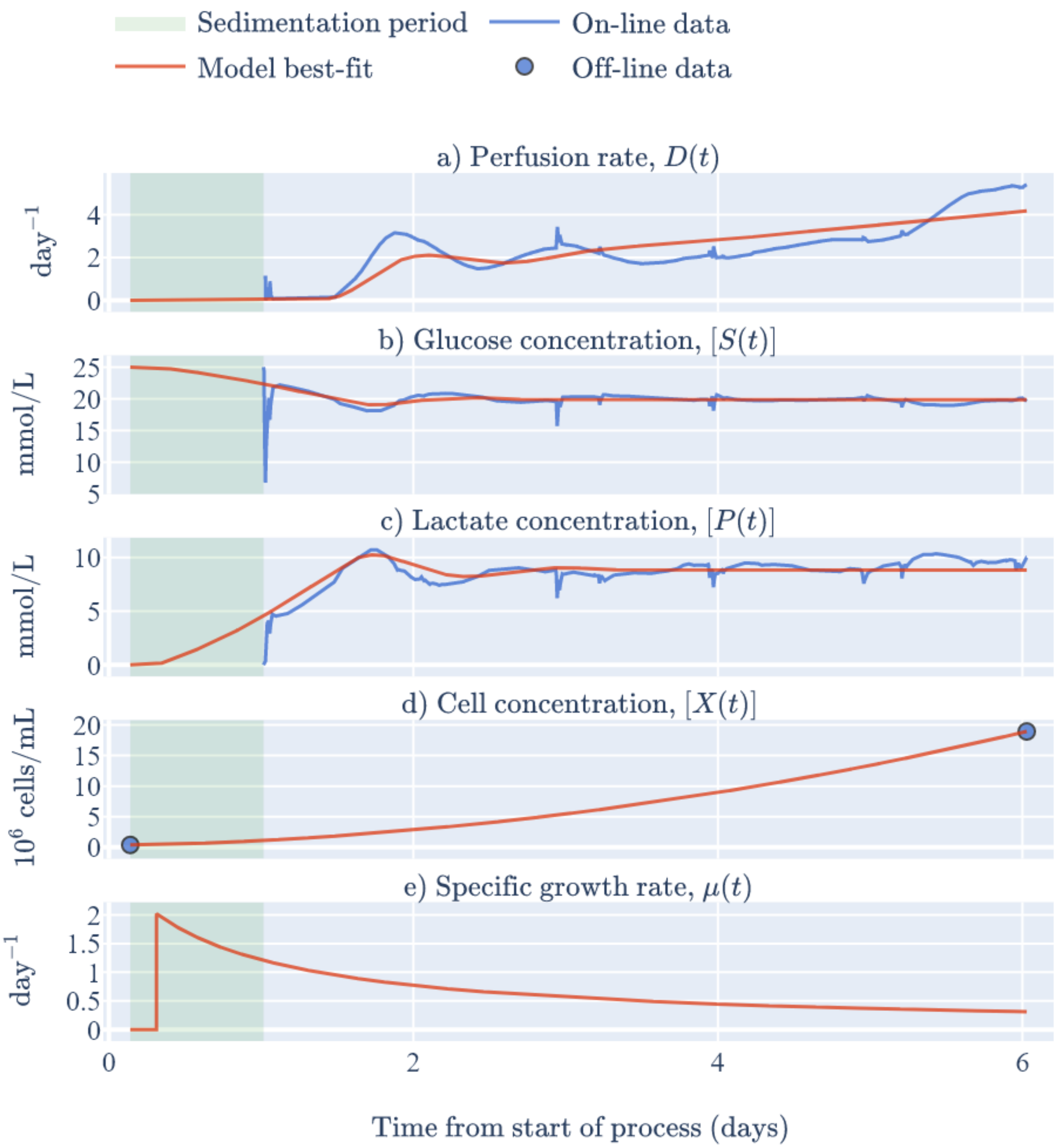

Figure S1. Key data from run 2 in the larger chamber of the Aglaris FACER and model best-fits.

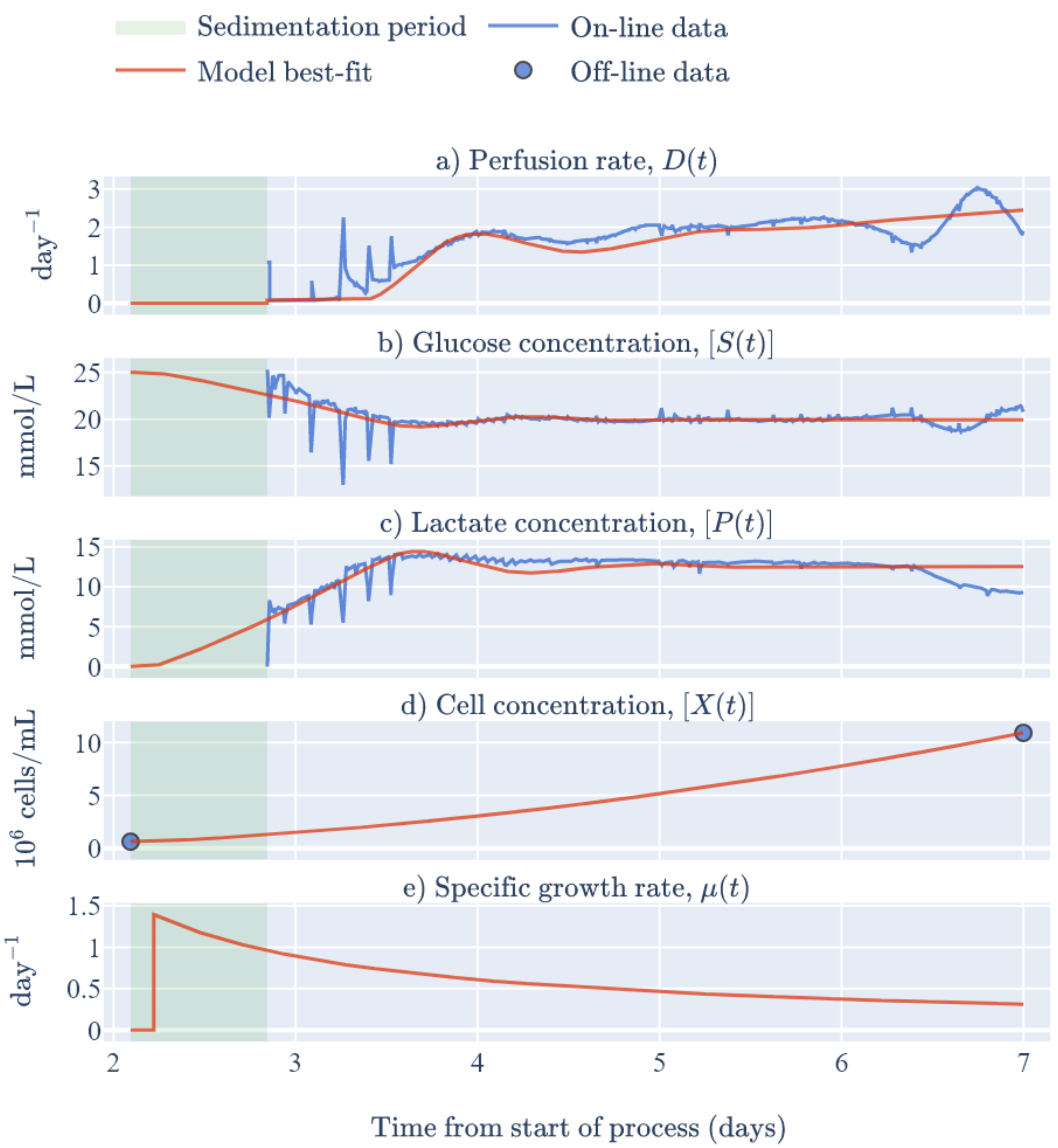

Figure S2. Key data from run 4 in the larger chamber of the Aglaris FACER and model best-fits.

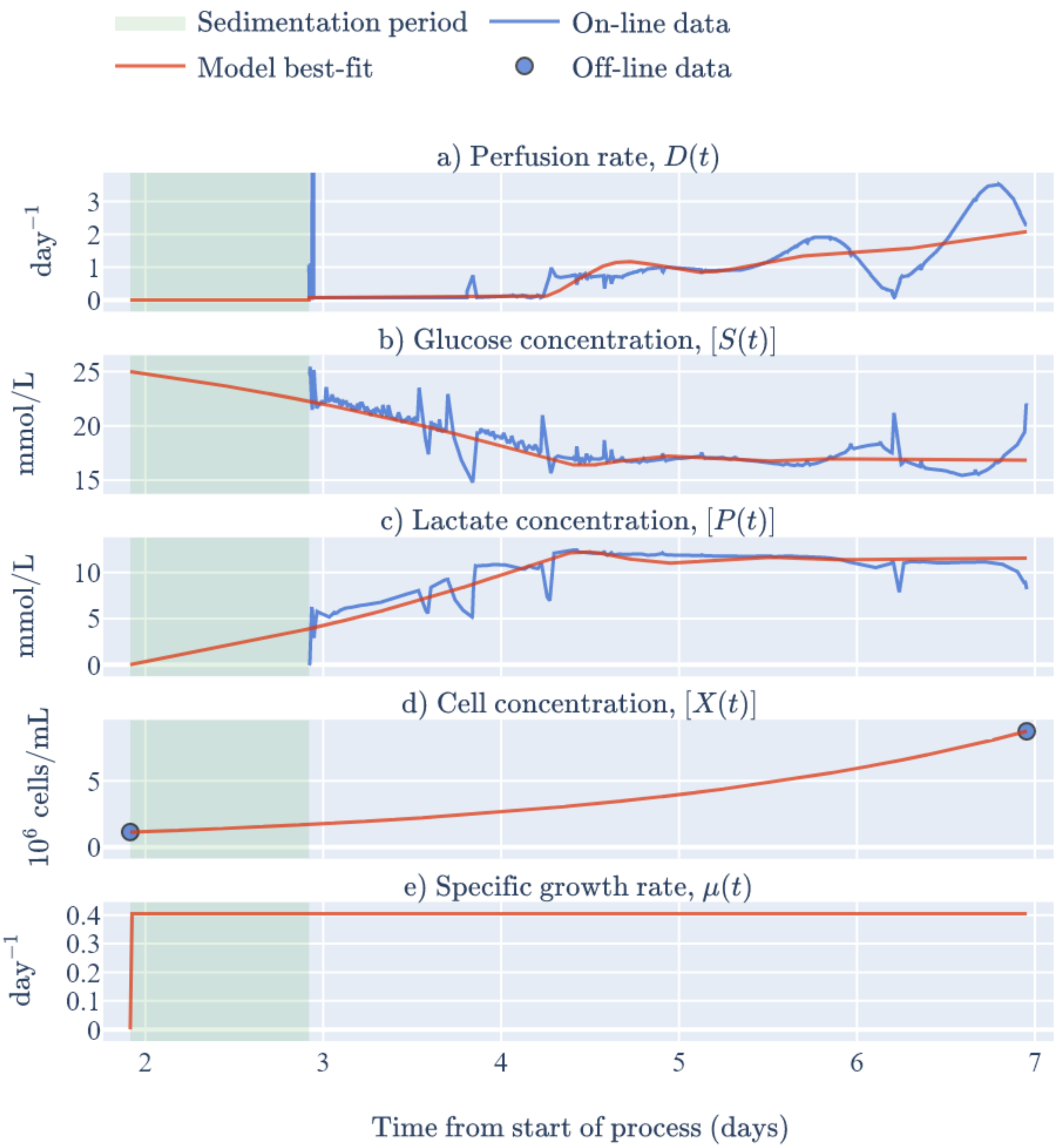

Figure S3. Key data from run 5 in the larger chamber of the Aglaris FACER and model best-fits.

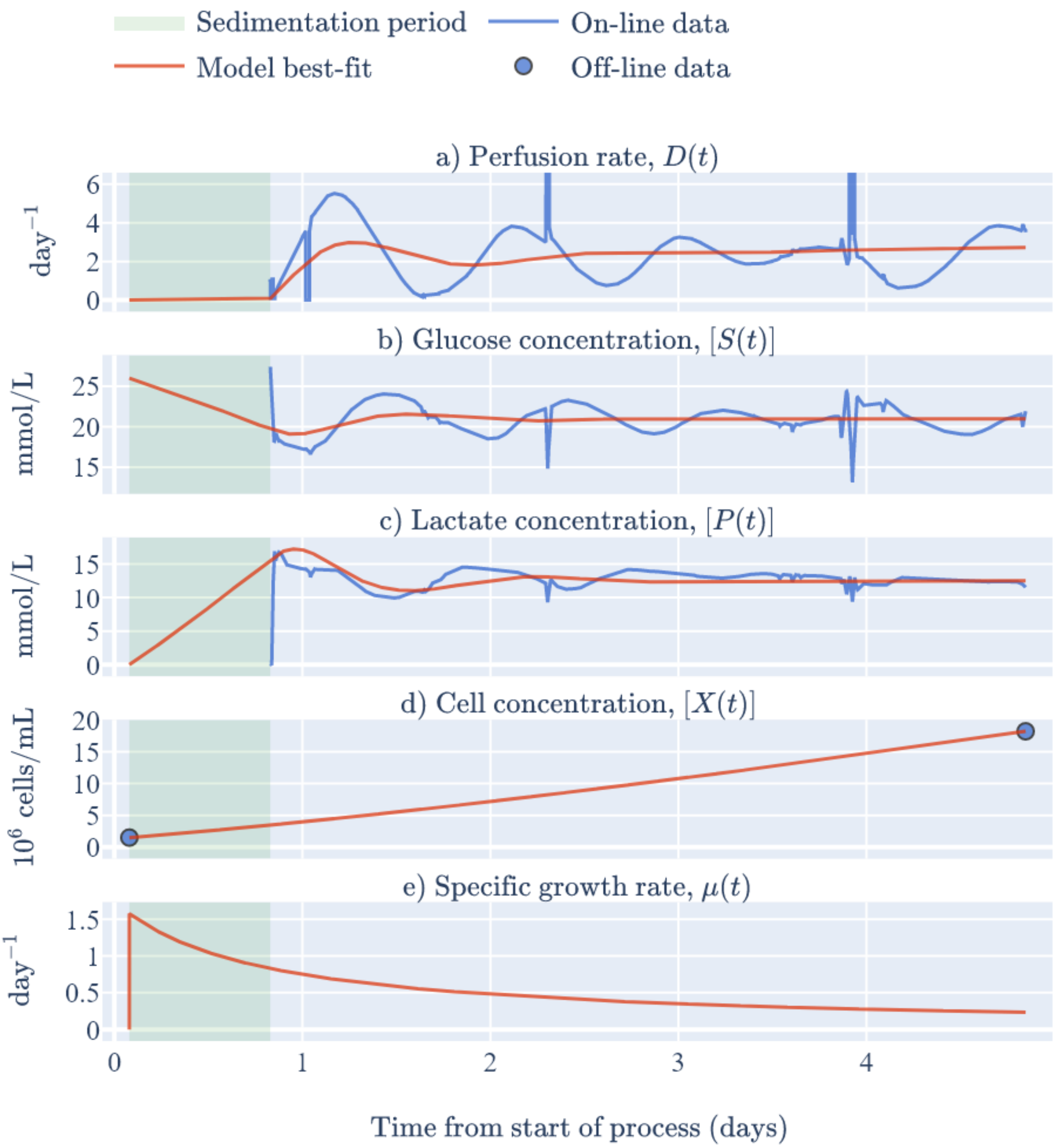

Figure S4. Key data from run 6 in the larger chamber of the Aglaris FACER and model best-fits.

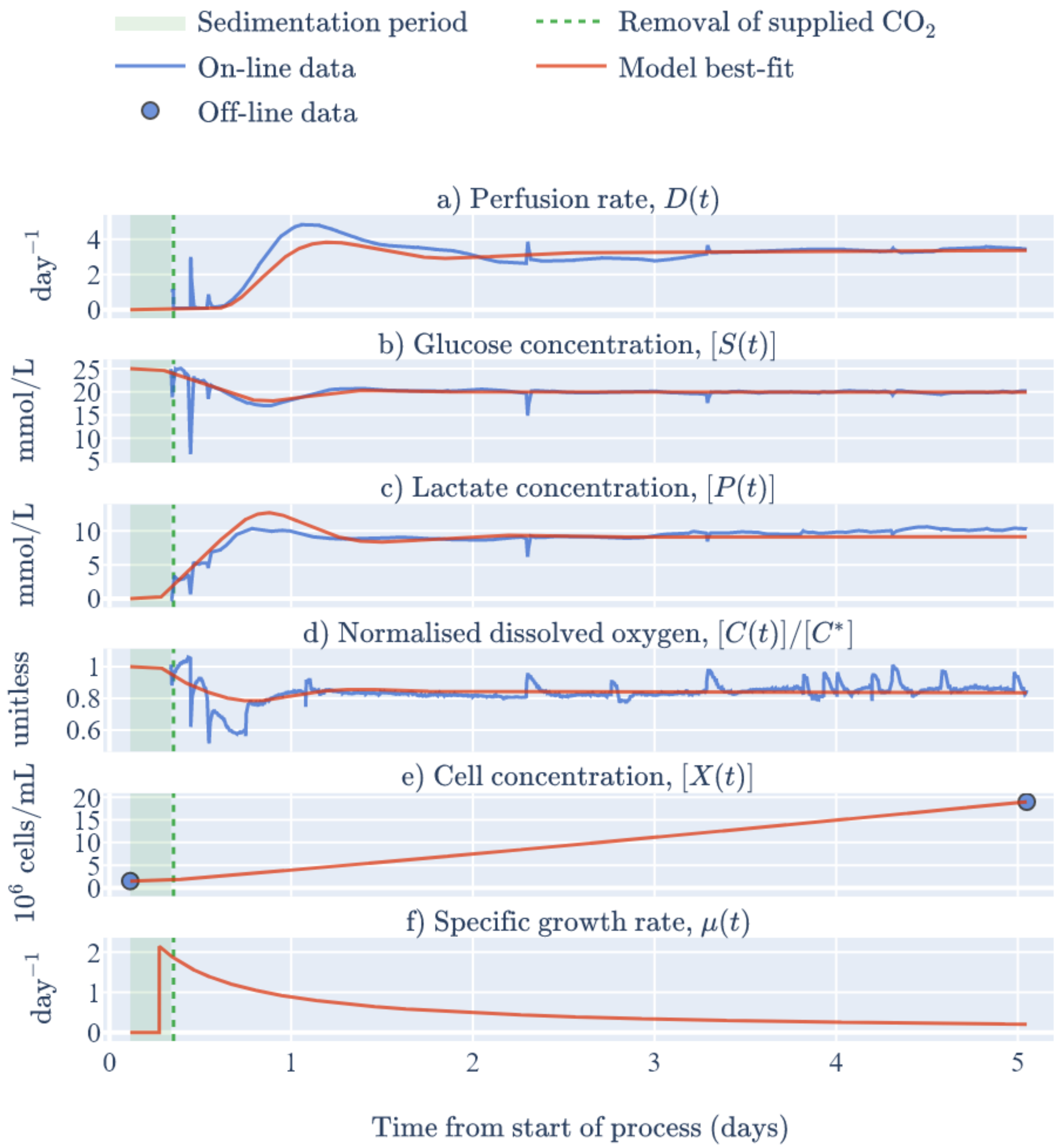

Figure S5. Key data from run 1 in the larger chamber of the Aglaris FACER and best-fits of extended model that additionally fits to the normalized DO data.

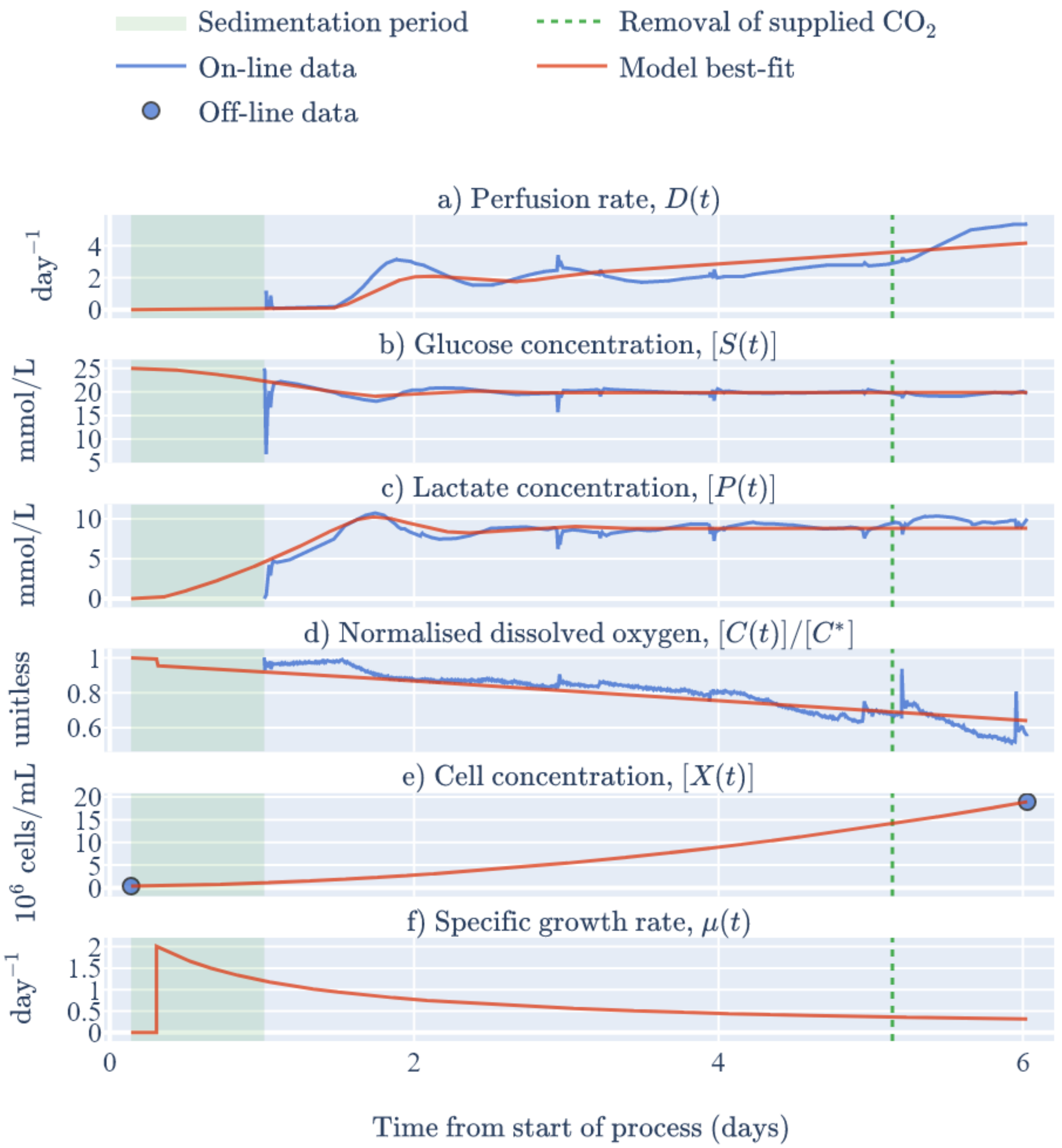

Figure S6. Key data from run 2 in the larger chamber of the Aglaris FACER and best-fits of extended model that additionally fits to the normalized DO data.

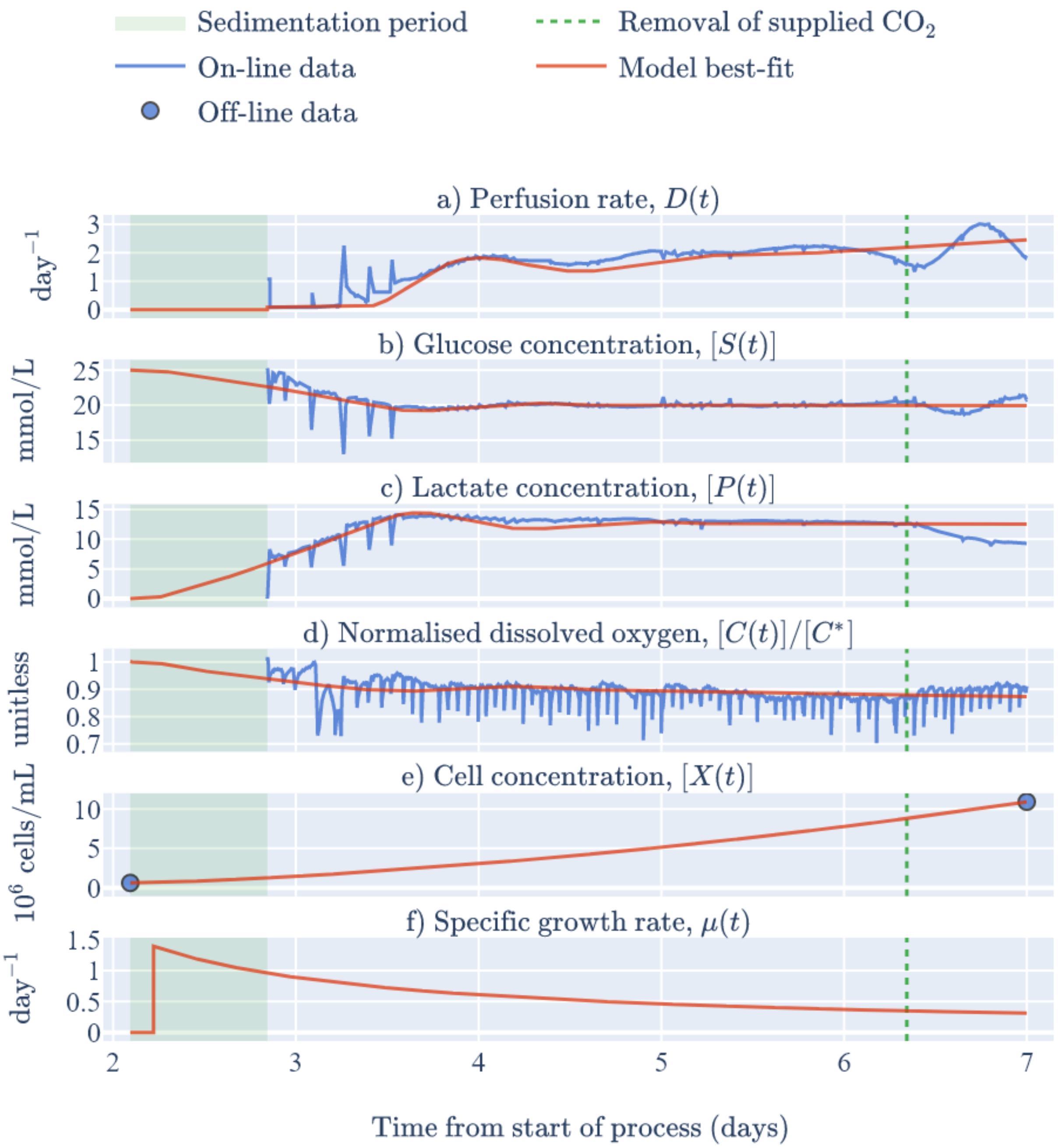

Figure S7. Key data from run 4 in the larger chamber of the Aglaris FACER and best-fits of extended model that additionally fits to the normalized DO data.

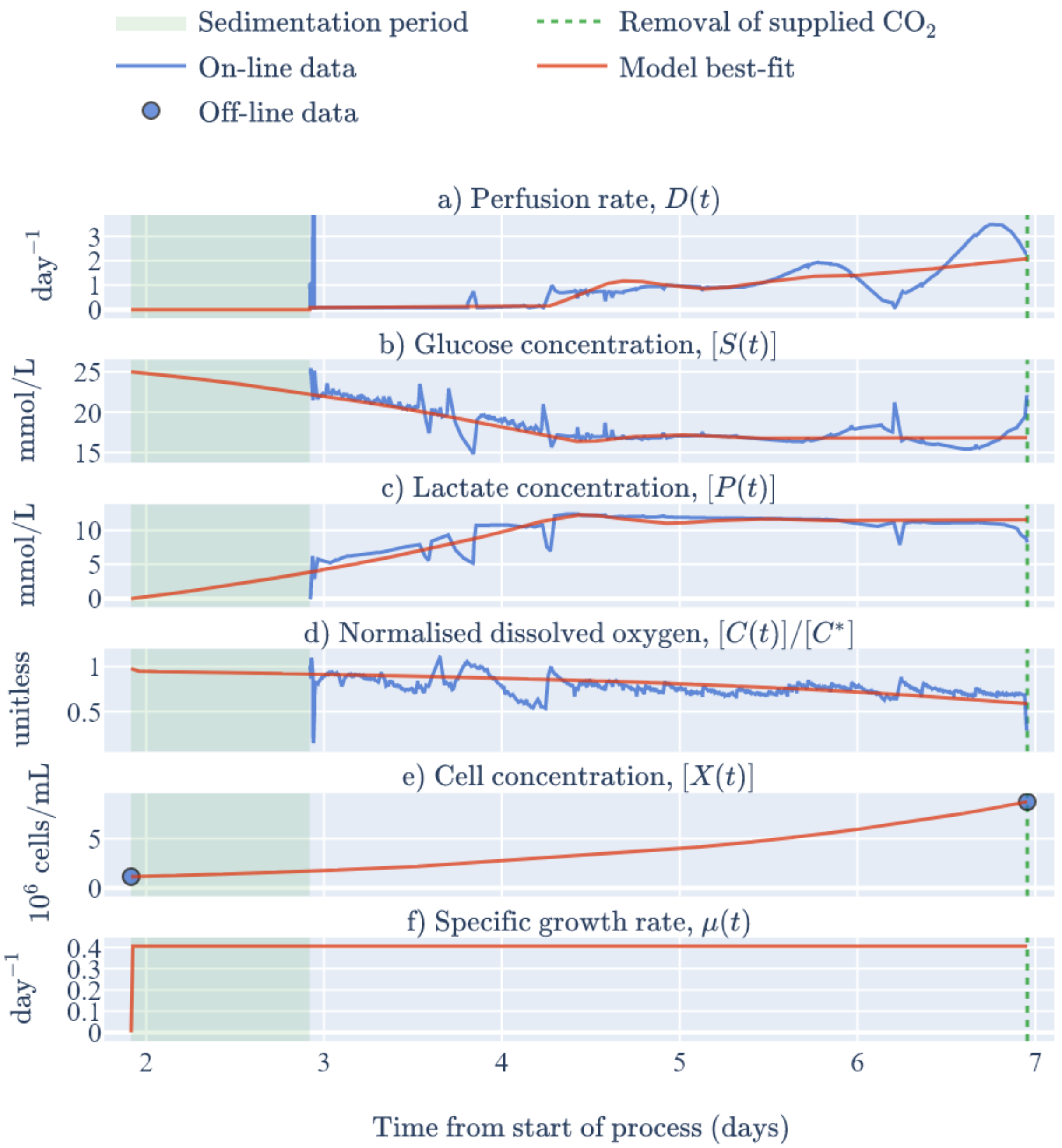

Figure S8. Key data from run 5 in the larger chamber of the Aglaris FACER and best-fits of extended model that additionally fits to the normalized DO data.

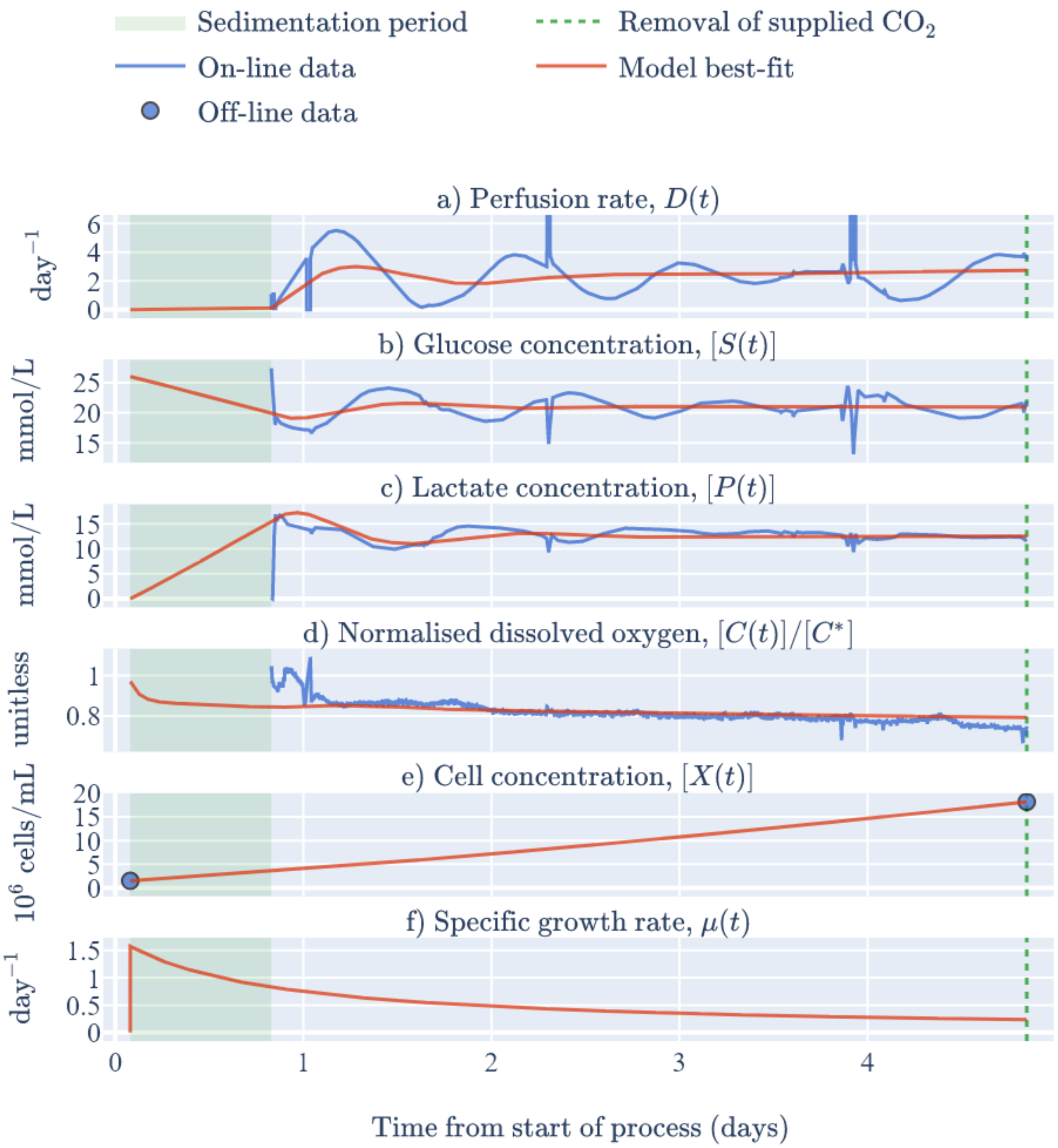

Figure S9. Key data from run 6 in the larger chamber of the Aglaris FACER and best-fits of extended model that additionally fits to the normalized DO data.

REFERENCES

[1]F. J. Richards. A flexible growth function for empirical use. *Journal of Experimental Botany*, 10(2):290–301, 1959.

[2]Shijie Liu. *Bioprocess Engineering: Kinetics, Sustainability, and Reactor Design*. Elsevier, third edition, 2020.

[3]Even Tjørve and Kathleen M.C. Tjørve. A unified approach to the Richards-model family for use in growth analyses: Why we need only two model forms. *Journal of Theoretical Biology*, 267(3):417–425, 2010.

[4]John Wolberg. *Data Analysis Using the Method of Least Squares: Extracting the Most Information from Experiments*. Springer Berlin, Heidelberg, first edition, 2006.

[5]Matthew Newville, Till Stensitzki, Daniel B. Allen, and Antonino Ingargiola. LMFIT: Non-Linear Least-Square Minimization and Curve-Fitting for Python (0.8.0), 2014.

[6]David L.I. Janzén, Linnéa Bergenholm, Mats Jirstrand, Joanna Parkinson, James Yates, Neil D. Evans, and Michael J. Chappell. Parameter identifiability of fundamental pharmacodynamic models. *Frontiers in Physiology*, 7(DEC):1–12, 2016.

[7]Milena Anguelova, Johan Karlsson, and Mats Jirstrand. Minimal output sets for identifiability. *Mathematical Biosciences*, 239(1):139–153, 2012.

[8]Andreas Raue, Johan Karlsson, Maria Pia Saccomani, Mats Jirstrand, and Jens Timmer. Comparison of approaches for parameter identifiability analysis of biological systems. *Bioinformatics*, 30(10):1440–1448, 2014.

[9]Wolfram Research Inc. Mathematica, 2023.
